# Evaluation and comparison of most prevalent artifact reduction methods for EEG acquired simultaneously with fMRI

**DOI:** 10.3389/fnimg.2022.968363

**Published:** 2022-08-29

**Authors:** Aleksij Kraljič, Andraž Matkovič, Nina Purg, Jure Demšar, Grega Repovš

**Affiliations:** ^1^Department of Psychology, Faculty of Arts, University of Ljubljana, Ljubljana, Slovenia; ^2^Faculty of Computer and Information Science, University of Ljubljana, Ljubljana, Slovenia

**Keywords:** EEG-fMRI, carbon-wire loop, ballistocardiogram, MR artifacts, EEG preprocessing, imaging artifact, Bayesian statistics in neuroscience

## Abstract

Multimodal neuroimaging using EEG and fMRI provides deeper insights into brain function by improving the spatial and temporal resolution of the acquired data. However, simultaneous EEG-fMRI inevitably compromises the quality of the EEG and fMRI signals due to the high degree of interaction between the two systems. Fluctuations in the magnetic flux flowing through the participant and the EEG system, whether due to movement within the magnetic field of the scanner or to changes in magnetic field strength, induce electrical potentials in the EEG recordings that mask the much weaker electrical activity of the neuronal populations. A number of different methods have been proposed to reduce MR artifacts. We present an overview of the most commonly used methods and an evaluation of the methods using three sets of diverse EEG data. We limited the evaluation to open-access and easy-to-use methods and a reference signal regression method using a set of six carbon-wire loops (CWL), which allowed evaluation of their added value. The evaluation was performed by comparing EEG signals recorded outside the MRI scanner with artifact-corrected EEG signals recorded simultaneously with fMRI. To quantify and evaluate the quality of artifact reduction methods in terms of the spectral content of the signal, we analyzed changes in oscillatory activity during a resting-state and a finger tapping motor task. The quality of artifact reduction in the time domain was assessed using data collected during a visual stimulation task. In the study we utilized hierarchical Bayesian probabilistic modeling for statistical inference and observed significant differences between the evaluated methods in the success of artifact reduction and associated signal quality in both the frequency and time domains. In particular, the CWL system proved superior to the other methods evaluated in improving spectral contrast in the alpha and beta bands and in recovering visual evoked responses. Based on the results of the evaluation study, we proposed guidelines for selecting the optimal method for MR artifact reduction.

## 1. Introduction

The use of electroencephalography (EEG) and functional magnetic resonance imaging (fMRI) has enabled significant advances in our understanding of brain and cognition in health and disease. Each of the two methods brings a number of advantages and disadvantages. EEG enables tracking changes in electrical brain potentials that directly reflect neuronal activity with high temporal precision. The observed activity is, however, difficult to localize because the signal can only be measured at sparse points on the scalp and the volume conduction of the human head further degrades the precision of localizing the neuronal sources contributing to the recorded electrical activity. Modern fMRI allows functional brain imaging with millimeter spatial precision. However, the blood-oxygen-level-dependent (BOLD) signal is only an indirect measure of neuronal activity, which is further smoothed in time due to the nature of the hemodynamic response, resulting in poor temporal precision. The limitation of each modality used alone can be overcome by combining the results obtained using both methods even when the data is acquired separately. For instance, results of fMRI can provide constraints or informative priors for EEG source analysis, whereas precise EEG timing can supplement the results of fMRI analyses. However, even further benefits can be achieved by simultaneous EEG-fMRI recording as in this case the recorded signals reflect the same neuronal events. This concurrency increases the validity of data fusion and enables additional types of analyses. Additionally, the signals from each of the two modalities reflect distinct features of neuronal responses and can present correlates for the other modality (Bin He and Zhongming Liu, 2008).

Although data fusion allows more accurate inferences about the brain activity being studied, it also comes at a cost of detrimental interactions between the two systems. The presence of EEG equipment inside the MRI scanner room may affect the homogeneity of the magnetic field and lead to signal loss in the BOLD signal (Krakow et al., 2000; Mullinger et al., 2008). On the other hand, as described in Faraday's law of induction, changes in magnetic flux through a conductive loop, whether due to movements of the conductor or alterations of the magnetic field, induce electric currents inside a conductor (Young et al., 2008). Both occur during fMRI recordings, which require a strong static magnetic field and rapid changes in magnetic field strength during image acquisition, resulting in electromagnetic induction in the conductive loops formed by the collection of EEG electrodes and the complex conductive structure of human head tissue. The resulting MR artifacts are superimposed on the neuronal signal measured by the EEG system and generally exceed it in amplitude. A variety of methods have been proposed to reduce MR artifacts in EEG signals, but few of them seem to dominate in the EEG-fMRI research community (Bullock et al., 2021). This is most likely due to the fact that only a subset of accepted methods are readily available in the widespread EEG processing toolboxes.

To select an optimal strategy for dealing with MR artifacts in EEG data, we designed a study to empirically evaluate a number of the most commonly used and readily available artifact reduction methods. Because different artifact reduction methods do not always perform equally well on different types of EEG data analyses, we performed the evaluation on three different EEG paradigms with known and robust temporal and spectral neuronal responses. For a direct comparison of MR artifact reduction methods, we collected data from each participant on all three tasks both simultaneously with fMRI acquisition in the MR scanner and outside the MRI environment. The recording outside the scanner served as a benchmark for evaluating artifact reduction results. The statistical inference in the evaluation of artifact reduction methods was conducted using hierarchical Bayesian probabilistic modeling.

In addition to computational artifact reduction methods, we also evaluated a method using a reference signal recorded with a set of six carbon-wire loops (CWL) (van der Meer et al., 2016), which are isolated from the scalp and exclusively capture MR-induced artifacts. The CWL method was chosen because it is affordable, straightforward to implement, and the corresponding software is openly available. This addition allowed us to assess the benefits of MR artifact reduction utilizing reference signals and to identify the types of studies in which its use could play a critical role in the effort to obtain high-quality EEG data.

In the following sections, we first introduce the three main types of MR artifacts and their characteristics, and then briefly review the most common and available artifact reduction methods before outlining our specific aims and goals. For a systematic review of MR artifact reduction methods, please see Bullock et al. (2021).

### 1.1. Properties of MR artifacts

The two main mechanisms leading to magnetic flux changes are constantly present during simultaneous EEG-fMRI scanning. First, gradient and radiofrequency (RF) coils alter the magnetic field during each MRI acquisition, resulting in an undesirable artifact known as gradient or imaging artifact. Second, movements of the human head and EEG cables occur due to the vibrations of the MRI scanner as well as voluntary and involuntary movements (Allen et al., 1998; van der Meer et al., 2016). Voluntary movements should be minimized, however, involuntary movements of the head and electrodes arising from ballistic cardiac activity cannot be prevented. They result in a distinct MR artifact called ballistocardiogram (BCG) artifact.

**Figure 1:**
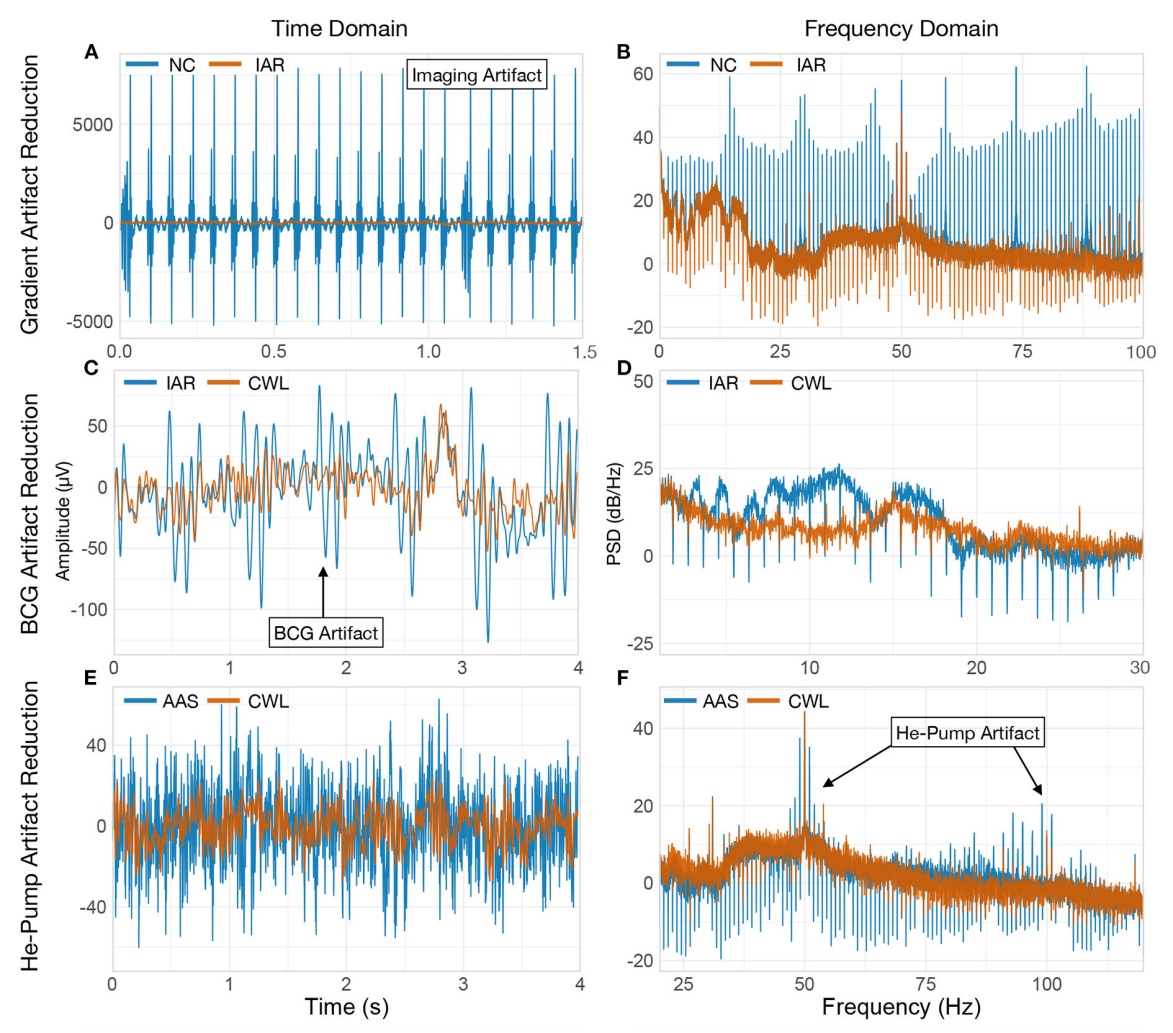
MR artifact properties. Single channel (Fp1) EEG signal of one participant illustrating the effects of three types of MR artifacts. Power spectral densities (PSD) were estimated with Welch's method (Welch, 1967) with a 2^15^ samples long Hamming window and a 50% window overlap. **(A,B)** MR imaging artifact is evident on the NC signal when shown along with the IAR signal in time and frequency domains. In the frequency domain NC signal exhibits high peaks at MRI slice acquisition frequency harmonics. IAR signal shows strong signal attenuation at frequencies equal to the reciprocal of the artifact template length. This is an expected consequence of fixed-length template subtraction. **(C,D)** BCG artifact present in the IAR signal is shown along BCG-corrected signal (using CWL) in the time and frequency domain. Signal in the time domain was low-pass filtered at 35 Hz for clarity. **(E)** Helium pump artifact contamination can be seen in the signal that underwent IAR and AAS for BCG correction (AAS), when compared to signal that was cleaned using CWL. **(F)** Helium pump artifact harmonics can be seen in the two indicated regions of the spectrum. NC, non-corrected signal; IAR, data with the imaging artifact reduced; AAS, data with IAR and reduced BCG artifacts with average artifact subtraction; CWL, data with IAR and carbon-wire loop artifact correction.

Imaging artifacts arise primarily from changes in the magnetic field due to rapid switching of gradient coils for spatial encoding during echo-planar imaging (EPI) and RF pulses for proton spin excitation (Mulert and Lemieux, 2010). The amplitude of the imaging artifact can be as high as a few hundred millivolts (mV), but due to the large amount of artifact's energy residing above the frequency range of the amplifier, the recorded imaging artifacts typically reach up to a few tens of mV (Allen et al., 2000). Despite the very high amplitudes, the precisely preprogrammed gradient coils switching sequence inducing the imaging artifact is expressed with a deterministic and periodic shape, dominated by harmonics of the slice repetition frequency convolved with harmonics of the volume repetition frequency (see Figures 1A,B) (Mandelkow et al., 2006; Ritter et al., 2007).

BCG artifacts originate from ballistic and nonballistic effects of cardiovascular activity in interaction with the magnetic field (Mulert and Lemieux, 2010). A detailed analysis of BCG mechanics is provided by Debener et al. (2008), who attribute the induction of the BCG to nodding head rotation, due to axial blood flow momentum and movements due to expansion of electrodes located near larger blood vessels (Debener et al., 2008; Mullinger et al., 2013). A nonballistic factor contributing to BCG has been attributed to the potential difference across blood vessels in the presence of a magnetic field, known as the Hall effect (Müri et al., 1998).

The magnitude of the BCG artifact depends directly on the strength of the static magnetic field of the scanner (Debener et al., 2008). It typically exceeds 50 μV (see Figure 1C) in 3 T scanners (Allen et al., 1998), while most of its power is contained below 25 Hz (see Figure 1D) and overlaps with the neuronal electrical activity of interest (Debener et al., 2007). Because BCG originates from electromagnetic induction caused by cardiac activity, it exhibits a periodic shape in sync with the heart rate.

In addition to imaging and BCG artifacts, vibrations of the human head and EEG cables originating from the scanner also lead to the induction of periodic artifacts. The most prominent source of vibration artifacts is the liquid helium pump of the MRI cooling system, while other sources such as the ventilation system may also contribute to some extent (Nierhaus et al., 2013). Vibration artifacts are scanner-dependent, but generally reside at higher frequencies (≥ 30 Hz) (see Figures 1E,F). This allows researchers not interested in high-frequency EEG to simply filter the signals with a low-pass filter. The artifact is often avoided altogether by simply turning off the helium pump during fMRI acquisition. Although this is a viable solution, some facilities prohibit operators from turning off the helium pump cooling system because it could have adverse effects on the MRI scanner.

### 1.2. MR artifact reduction methods

The periodicity and deterministic shape of the imaging artifact make the process of artifact reduction much more straightforward than reducing other MR artifacts. The first effective and most widely used algorithm for imaging artifact reduction (IAR), called average artifact subtraction (AAS), was proposed by Allen et al. (2000). In this method, the EEG signal is averaged (channel-wise) over several fMRI volume or slice periods to create an artifact template, which is then subtracted from the raw EEG signal.

AAS assumes that the EEG signal of interest is uncorrelated between the epochs averaged when computing the average artifact template and that the induced imaging artifact is stationary over time (Hill et al., 1999). The first assumption can be violated by averaging the signal with a period matching that of the electrical neuronal activity (Allen et al., 2000). The second assumption is violated whenever the participant moves, changing the morphology of the induced artifact. Since the assumptions do not always hold in practice, some residual artifacts remain in the EEG signal. Another important source of residual artifacts in AAS is the imperfect temporal alignment of slice or volume markers, which depends on the quality of synchronization of the clocks of the EEG and MRI systems.

Out of many different approaches published for IAR, many present variations of AAS. The main differences between them are either in modifications in the computation of the artifact template and/or the inclusion of additional steps for reducing residual artifacts. For instance, adaptive noise cancellation (ANC) by Allen et al. (2000) performs additional filtering of signal components correlated with the MRI slice/volume frequency after AAS, FMRI Artifact Slice Template Removal (FASTR) by Niazy et al. (2005) utilizes principal component analysis (PCA) and ANC after AAS, and Realignment parameter informed artifact correction by Moosmann et al. (2009) exploits the additional realignment information from MRI images for better selection of imaging artifact epochs in AAS template computation.

Due to the periodic nature of the BCG artifact, most approaches to BCG reduction, first proposed by Allen et al. (1998), were initially based on AAS. However, due to a larger degree of violation of the stationarity assumption of the process underlying these artifacts, AAS proved to be less effective for correcting BCG artifacts than for IAR (Niazy et al., 2005). This motivated the development of more advanced BCG reduction methods. Niazy et al. (2005) and Negishi et al. (2004) proposed a method for estimating BCG artifact templates from temporal principal components, the optimal basis set (OBS). OBS uses PCA to compute template artifact shape for each channel independently. The template is then fitted and subtracted from each artifact occurrence (Niazy et al., 2005).

The assumption that cardiac and neuronal activity are independent has led many researchers to exploit the capabilities of independent component analysis (ICA) (Srivastava et al., 2005). Reported evaluations led to different conclusions about the effectiveness of ICA in reducing BCG artifacts, suggesting that its performance is highly sensitive to the specific ICA algorithm used, parameter selection, and approach to classification of BCG-related independent components (Vanderperren et al., 2007; Liu et al., 2012). ICA was also sometimes combined with AAS or OBS to remove residual artifacts (Debener et al., 2007).

Recent state-of-the-art methods for MR artifact reduction utilize reference signals that capture induced artifact potentials without neuronal EEG. Reference signal systems are implemented either as a separate reference-layer of electrodes isolated from the scalp (Chowdhury et al., 2014; Steyrl et al., 2017) or as a set of six CWLs (four loops on participants' heads, two on EEG cables) (Masterton et al., 2007; van der Meer et al., 2016). Reference signals contain exclusively MR-induced electrical potentials because they are isolated from the scalp, as such, they are used for modeling BCG and other motion-related artifact templates to be subtracted from EEG signal. Particularly in CWL regression, a general linear model approach is used. The signals from the six carbon-wire loops and their time-shifted copies are regressed out of the EEG signal. The procedure is performed for each channel independently, iteratively across individual time windows (for details see van der Meer et al., 2016).

Reference signal methods have shown superior performance in complete MR artifact reduction (Chowdhury et al., 2014; Hermans et al., 2016; Steyrl et al., 2017) and in BCG and helium pump artifact reduction van der Meer et al. (2016). The disadvantage of the second layer of electrodes is the complexity of the system, especially for high-density EEG caps, because the system needs to acquire twice the amount of data and the additional set of electrodes doubles the number of leads and amplifiers. These problems are avoided by the CWL system, which requires minimal additions to the acquisition setup, albeit at the price of slightly reduced performance (Hermans et al., 2016).

All MR artifact correction methods will somewhat adversely affect the signal, whether by introducing additional distortions or by removing some information of interest. For this reason, it is necessary to be confident that the preprocessing methods used have the least possible impact on the analyzed signal. It is therefore essential to take preventive measures and carefully select data acquisition settings during an EEG-fMRI experiment to reduce the amplitude of induced artifacts and achieve better artifact reduction results during signal processing.

### 1.3. Specific aims and goals

The main aim of this evaluation study was to provide practical information to researchers working with simultaneously recorded EEG-fMRI to help them design an experimental setup and choose an artifact reduction method that fits their task design, research questions, and analyses of interest. Specifically, our goals were to (i) review the current literature and available tools to identify the most widely used, freely available, and easy-to-use methods for MR artifact reduction, (ii) evaluate their performance on example datasets covering a spectrum of EEG analyses, and (iii) suggest the optimal MR artifact reduction pipeline for different analysis goals.

Based on the review presented above, we decided to use AAS as the method for imaging artifact reduction, because it is readily available through various toolboxes and it was reported to be more conservative in terms of additionally reducing other types of artifacts (Bullock et al., 2021). Next, we identified AAS and OBS as the two main BCG artifact reduction methods of interest. Both are widely used and have been often reported to yield similar performance (Bullock et al., 2021). Some sources, however, report conflicting results regarding their effectiveness (Bullock et al., 2021). We decided to reevaluate both methods on our dataset to investigate whether either method performed better in a given setting. Lastly, several studies (Hermans et al., 2016; van der Meer et al., 2016) indicated that the CWL approach is superior to and outperforms other MR artifact reduction methods, but the scope of testing was rather limited in terms of the diversity of paradigms on which evaluations were performed. To help researchers decide whether to invest in a CWL system for their future studies, we included a CWL system in our evaluation to assess its advantages over more conventional approaches in contexts where the method has not yet been evaluated.

To cover a variety of study designs and analyses, we focused our data collection on three behavioral paradigms. First, a resting-state task with eyes-open (EO) and eyes-closed (EC) conditions allowed assessment of the effect of MR artifact reduction methods on continuous EEG signal analysis and an assessment of the ability to resolve frequently reported changes in alpha band power. Second, we employed a finger tapping paradigm to evaluate the effect of MR artifact reduction methods on task-related (de)synchronization in the alpha, beta, and gamma frequency bands. Lastly, we used visual evoked potentials (VEP) to evaluate the impact of the MR artifact reduction methods on event-related potentials (ERP) analyses.

## 2. Materials and methods

### 2.1. Participants

Sixteen healthy young adults (10 women, 18–23 years old) participated in the evaluation study that included a simultaneous EEG-fMRI session and a stand-alone EEG session outside the MRI environment. Of the 16 participants, only one was left-handed and was excluded from the finger tapping analysis. Due to various problems during the recording sessions, we excluded additional participants from specific analyses. Specifically, one participant experienced discomfort and only completed the resting-state task, data from some participants were missing either task or MR markers, while for one participant we failed to collect CWL data. As a result, we included 12 participants in the resting-state and VEP task analysis and 13 participants in the finger tapping task analysis. The study was approved by the Ethics Committee of the Faculty of Arts, University of Ljubljana, Slovenia. All participants gave written informed consent prior to participating in the study.

### 2.2. Behavioral tasks

The study included three tasks: (i) a resting-state task, (ii) a finger tapping task, and (iii) a checkerboard visual stimulation task. Participants performed all three tasks twice. First, only EEG data were acquired outside the MRI environment, then the same tasks were performed during simultaneous fMRI acquisition. All tasks were implemented in PsychoPy3 (Peirce, 2007).

***Resting-state task*** consisted of 22 alternating 40-s eyes-open (EO) and eyes-closed (EC) blocks. The beginning and end of each block were signaled by a brief high-pitched tone and visual instructions on the screen, followed by a 5-s pause. To avoid the inclusion of sensory evoked potentials, the pause was not included in the analysis.

In the ***finger tapping task*** (Ball et al., 2008; Darvas et al., 2010), participants were instructed to tap the fingers of the right hand in 3-s blocks followed by a 3-, 5-, or 7-s rest (i.e., no hand movement). Tapping consisted of touching a thumb and a finger in the following order: index, ring, middle, and little finger. Each tapping block was signaled by the image of a hand on a screen. During rest blocks, the screen was blank. Participants completed 60 tapping blocks.

In the ***checkerboard visual stimulation task***, participants were shown an image of a ring-shaped radial checkerboard pattern in one of the four quadrants of a visual field at each trial (LU, left upper; LL, left lower; RU, right upper; RL, right lower) (Capilla et al., 2016). The pattern spanned 80° of a full circle and 13° of visual angle. The checkerboard pattern consisted of 2.5 angular cycles (5 "spokes") and 4 radial cycles (8 “rings”), of which only the outer 3 were shown. Each stimulus was shown for 700 ms. The intertrial interval (ITI) was randomly chosen between 1.7, 3.7, and 5.7 s. There were 42 trials in each condition. The order of trials was randomized. To ensure that participants were attending to the task, they were instructed to press a right arrow key on a response pad with their right middle finger when a pattern was displayed on the right part of the screen or a left arrow key with their right index finger when a pattern was displayed on the left part of the screen. Incorrect trials were not excluded from the analysis. Participants were instructed to always look at the fixation cross in the center of the screen.

### 2.3. Data acquisition

#### 2.3.1. fMRI data acquisition

MRI data were acquired using the Philips Achieva 3.0T TX scanner. BOLD images were acquired with T2*-weighted echo-planar imaging sequence (3 BOLD images, field of view = 221 × 221 mm, 64 axial slices, voxel size = 2.3 × 2.3 × 2.3 mm, matrix = 96 × 95, TR = 1, 100 ms, TE = 27 ms, flip angle = 52°, MultiBand SENSE factor 4, SENSE P reduction 1.4, 950 frames for the resting-state task, 605 for the checkerboard task, and 620 for the finger tapping task). Due to internal regulations of the MRI facility, we were not allowed to turn off the helium pump of the scanner's cryostat to avoid helium pump-induced MR artifacts in the EEG data.

#### 2.3.2. EEG data acquisition

EEG data were recorded outside the MRI environment and simultaneously with fMRI BOLD acquisition using an MR-compatible EEG system consisting of a 128-channel BrainCap-MR and a set of BrainAmp MR amplifiers (Brain Products GmbH, Gilching, Germany). The impedance of the electrodes was kept at approximately 15 kΩ. Data were recorded at a sampling rate of 5, 000 Hz and a resolution of ±0.5μV. Data were high-pass filtered with a time constant of 10 s and low-pass filtered with a cutoff frequency of 250 Hz at acquisition. The recorded signals of all electrodes were referenced to the vertex electrode (FCz). The EEG amplifiers and the MRI system clocks were synchronized with the Brain Products SyncBox system. The EEG amplifiers were placed outside the scanner bore so that the stimulus screen was not obscured. The CWL system consisted of six carbon wire loops, constructed as explained in van der Meer et al. (2016) (see Supplementary Figure S1), and its signals were recorded using a Brain Products BrainAmp ExG bipolar amplifier (±0.5μV resolution). In addition to task markers, we acquired TR markers sent by the MRI scanner at the beginning of each BOLD volume. The ECG signal was recorded with an additional ECG electrode placed on the participants' back below the left scapula.

### 2.4. EEG data preprocessing

#### 2.4.1. MR artifact reduction

MR artifact reduction was the first step in the EEG preprocessing pipeline, followed by other EEG preprocessing steps common to the majority of stand-alone EEG experiments. For the evaluation, we considered only methods that were openly and readily available for MATLAB (Mathworks, Natick, Massachusetts) and EEGLAB toolbox (Delorme and Makeig, 2004). In order to keep the complexity of the evaluation within reasonable limits, we decided to additionally limit the evaluation to parameters that are considered as default for the corresponding method.

***IAR:*** IAR is the first step in MR artifact reduction because it reduces the strongest artifact and prepares the signals for further artifact reduction of less deterministic artifacts, such as the BCG artifact and helium pump artifacts. To maintain central focus on evaluating the performance of different methods for reducing the more complex cardiac- and motion-related artifacts, we used a single IAR method. We considered FASTR as implemented through the fMRIb EEGLAB plug-in (Niazy et al., 2005) and AAS, as implemented through AMRI toolbox (Liu et al., 2012). Because it was suggested that the additional principal component removal (PCA) step performed by FASTR, on top of AAS and ANC, might lead to some BCG artifact reduction in addition to IAR (Bullock et al., 2021), we decided to use AAS from AMRI, which allowed us to independently evaluate IAR and BCG artifact reduction methods. For template computation, we used 25 averaging windows as suggested by Allen et al. (2000).

***BCG Artifact Reduction:*** For the BCG artifact reduction, we included the two most commonly used methods available for MATLAB, AAS and OBS, that are readily available through AMRI (Liu et al., 2012) and fMRIb (Niazy et al., 2005) toolboxes. AAS was used with the default 21 averaging windows. In OBS, as suggested by the authors, the first 3 principal components and the signal mean were included for BCG artifact fitting. Due to the uncertainties in using ICA for artifact reduction and the lack of readily available tools that would allow straightforward use of ICA, we decided not to include it in our evaluation. In particular, there are a large number of factors that must be considered when using ICA, and none of the automated pipelines are readily available. The first factor is the choice of ICA algorithm to use, and the second is the choice of algorithm and criteria for classifying the identified independent components as signal or noise (artifact). Several approaches were evaluated by Vanderperren et al. (2010), who suggested that ICA can achieve a similar level of BCG artifact reduction as OBS as well as additional residual artifact reduction when performed on top of OBS, but it does not generalize well and requires very careful monitoring of the process to avoid signal degradation.

***CWL Artifact Regression:*** CWL regression was performed using the cwleegfmri plug-in for EEGLAB (van der Meer et al., 2016). For the CWL regression, we chose default parameters, Hann window tapering function, window duration of 4 s, and delay of 0.021 s.

#### 2.4.2. Basic preprocessing

MR artifact reduction was followed by preprocessing, which was performed using EEGLAB (Delorme and Makeig, 2004), ERPLAB (Lopez-Calderon and Luck, 2014), and custom functions in MATLAB. To speed up further processing and reduce disk usage, the data were first downsampled to 500 Hz. Next, we performed epoching from −200 to 500 ms relative to stimulus presentation onset for the checkerboard task, from −3, 000 to 3, 000 ms in the case of the finger tapping task, and from 0 to 40 s for the resting-state task. Data were re-referenced to the average reference. For the checkerboard and finger-tapping tasks, epochs were manually inspected for large transient muscle artifacts and bad epochs were excluded from further processing. This was followed by manual channel inspection and exclusion of bad channels. In the checkerboard task, we performed adaptive mixture independent component analysis (AMICA) (Palmer et al., 2011) to remove eye movement and muscle-related artifacts. We manually classified independent components and interpolated previously excluded channels. Finally, we applied a low-pass filter with a cut-off frequency of 30 Hz for the resting-state and VEP data, and a low-pass filter with a cut-off frequency 100 Hz for the finger tapping task. Both low-pass filters were 683 samples long Hamming-windowed sync FIR filters. Filter length was estimated using Fred Harris' rule for desired stopband attenuation of 30 dB (Lyons, 2011). In a final step, the data were downsampled to 100 Hz for the checkerboard task and the resting-state, and to 250 Hz for the finger tapping task.

### 2.5. Evaluation data preparation

For each participant, we considered EEG data collected outside the MRI environment as a reference signal (REF) to which we compared five variants of the data collected in the MRI environment, each variant representing the signal after a different combination of artifact correction methods. The five variants were: NC, non-corrected data; IAR, data after removal of imaging artifacts using the AAS algorithm; OBS, data after IAR and additional OBS BCG artifact removal; AAS, data after IAR and additional AAS BCG artifact removal; CWL, data after IAR and additional CWL regression. All five variants were based on the same raw data, which allowed a direct comparison of the effectiveness of each preprocessing method.

#### 2.5.1. Spectral data extraction

Power spectral density (PSD) for all analyses of EEG spectral content was estimated using Welch's method (Welch, 1967) with a 2^12^ samples long Hamming window and a 50% window overlap. All estimated PSDs were converted to decibels (10log_10_). Working with data on the decibel scale also allowed us to model the outcome variables with normal distributions. Because of the short trial length, PSDs of finger tapping trials were computed on zero-padded data (2^12^−3, 000 = 1, 096 samples long padding). A mean PSD of multiple EEG channels (summary channels) was always computed by averaging the PSD of each channel, rather than averaging the signal in the time domain prior to the Fourier transformation.

As the first step of the resting-state occipital alpha analysis, we computed the grand-average PSD across the entire length (0–40 s) of the EO and EC block on every channel separately on REF data. Exclusively for the selection of channels for the resting-state data analysis, not for the statistical analysis, we computed the difference in PSD (ΔPSD) and averaged the PSD values in the broader alpha band (8–12 Hz) (Ritter et al., 2009) across all channel sites (see Figure 3A). Based on the standardized topography of the mean ΔPSD in the alpha band (see Figure 3B), we identified all channels that exceeded one standard deviation (selected channels were P3, P4, O1, O2, P7, P8, Oz, POz, PO3, PO4, P5, P6, PO7, PO8, PPO1h, PPO2h, POO1, POO2, PPO5h, PPO6h, TPP8h, PPO9h, PPO10h, POO9h, POO10h, PO9, PO10, and O10—highlighted in the topography in Figure 3B). These channels were used to compute a representative summary channel. The dataset used in the statistical analysis of the recovery of EC occipital alpha activity was derived by computing the mean PSD in the alpha band over every block in the data for each participant on the previously defined summary channel.

Data for finger tapping spectral analysis were prepared similarly to the data for the resting-state alpha analysis, except that we computed ΔPSD over each trial in three different frequency bands. In this case, we contrasted 3 s periods in which participants were instructed to remain still with 3 s periods in which they were instructed to perform the finger tapping pattern. The grand-average spectra for the alpha and beta bands were computed on the entire duration of the baseline (−3, 000–0 ms) and the finger tapping (0–3, 000 ms) periods, whereas the baseline spectra for the gamma band analysis were computed over the 1 s window prior to the instruction to begin tapping (−1, 000–0 ms) and the response spectra were computed over the 1 s window post the instruction (0–1, 000 ms). This was motivated by the results of Ball et al. (2008) and Darvas et al. (2010), where the largest gamma activity was observed around the onset of finger motion. From the two pairs of spectra, we computed their difference by subtracting the baseline PSD from the response PSD. The frequency region for the alpha band was defined the same as for the resting-state occipital alpha analysis in order to allow for comparison (8–12 Hz). For the beta and gamma bands, we defined the frequency region empirically based on the grand-average spectra of REF data, computed from a set of 12 left hemisphere central channel sites (C1, C3, C5, CP1, CP3, CP5, CCP1h, CCP3h, CCP5h, CPP1h, CPP3h, and CPP5h). Region boundaries were defined where pronounced activity was observed, which was 15–25 Hz for the beta band and 70–80 Hz for the gamma band. A narrower band around the peak was defined for the gamma activity since its response was much weaker compared to the alpha and beta activity. Once the frequency regions were defined, we extracted the mean power for each channel in all three bands and defined summary channels for each band from the standardized grand-average spectral difference topographies. We formed summary channels from the channels for which the mean power deviated from the mean by more than two standard deviations. For each band, we selected the following channels: (i) C3, CP1, C1, CP3, CCP3h for the alpha band, (ii) C3, CP1, C1, CP3, FCC3h, CCP3h, CCP5h for the beta band, and (iii) CP3, CCP3h for the gamma band (see Figure 4). The REF data spectrum computed on the summary channel defined for the alpha band shows the frequency regions for the alpha and beta bands (see Figure 4A). The spectrum computed on the summary channel for the gamma band is shown together with the defined frequency region in Figure 4B.

#### 2.5.2. Visual evoked potentials data extraction

To prepare the data for the ERP effect size analysis, we first computed the grand-average ERP of the ipsilateral and contralateral signals in the occipital region. The ipsilateral signal was computed as the mean signal across left hemisphere channels (all parietal and occipital channels on the left hemisphere) during the LL and LU stimuli and across right hemisphere channels (all parietal and occipital channels on the right hemisphere) during the RL and RU stimuli. The contralateral signal was computed as the mean signal over the left hemisphere channels during the RL and RU stimuli and over the right hemisphere channels during the LL and LU stimuli. We then computed a difference wave by subtracting the ipsilateral from the contralateral signal, which allowed us to identify two peaks in the early response period (≤ 300 ms). We used the two peaks to define two time windows of interest, D_1_ and D_2_, spanning 70–100 and 130–170 ms, respectively (see Figure 5A). Next, we computed the mean amplitude topography of the grand average signal in each of the two identified time windows of interest, separately for stimuli presented to the left and right visual fields (see Figure 5B), to identify the channels with the strongest VEP responses. To ensure the same level of noise attenuation on summary channels of both hemispheres due to channel averaging, we selected the same number of channels on each hemisphere. The number was determined as the average number of channels that exceeded two standard deviations from the mean on each hemisphere during each time window, thus we selected 7 channels with the largest positive amplitude during D_1_, and 7 channels with the largest negative amplitude during D_2_, from each hemisphere for each of the two time windows. The summary channels were computed as the mean signal from (i) P4, TPP8h, PO4, P8, P6, PO8, PPO6h for left stimuli on D_1_, (ii) PPO9h, P7, O1, PO3, P5, PPO5h, PO7 for right stimuli on D_1_, (iii) P6, PPO6h, P8, PO8, TPP8h, CPP6h, P4 for left stimuli on D_2_, and (iv) PPO5h, P5, PO7, TPP7h, P7, CPP5h, CP5 for right stimuli on D_2_ (see Figure 5B for their locations). For further statistical analysis of the effect size of the VEP difference between contralateral and ipsilateral stimuli, we extracted the mean amplitude across the identified channels in D_1_ and D_2_ windows of interest for each trial. We excluded the non-corrected (NC) data variant from all the analyses in the time domain due to the presence of imaging artifact components with very high amplitudes that dominated the neuronal responses and produced meaningless data for the evaluation.

To estimate the signal-to-noise ratio (SNR) for each of the variant datasets, we computed a single grand-average VEP across all trials (LL, LU, RL, RU) and the channels identified in the previous step. We then identified two prominent peaks in the signal, P_1_: 100–140 ms and N_1_: 150–200 ms, both of which corresponded with findings by Capilla et al. (2016). We computed two SNR estimates for P_1_ and N_1_ peaks. Trial-level SNR_t_ was computed across individual trials for each participant separately and then averaged, whereas participant-average ERP SNR_a_ was computed on the signal averaged over all trials from a participant. In both cases, SNR was estimated as the maximum root-mean-square (RMS) value in the time window of each of the two peaks divided by the mean RMS of the baseline signal (−200–0 ms) (Marino et al., 2018).

To compare the VEP responses in the reference data and each variant EEG-fMRI data sets, we first computed average VEP signals from 0 to 500 ms for each of the four stimulus conditions across the previously identified channels for each participant separately. We then estimated the similarity of the VEP responses by computing robust correlations (see Supplementary Methods 1.1.4) between the reference and variant time series, and the differences by computing the root-mean-square deviations (RMSD) between the reference and variant time series.

### 2.6. Statistical inference

**Figure 2:**
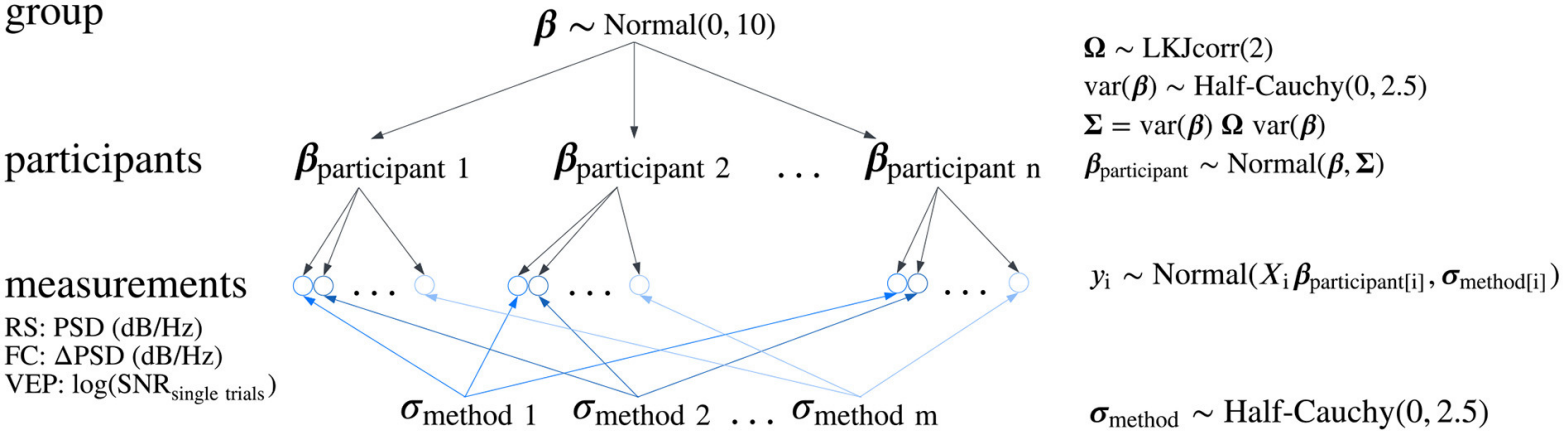
Hierarchical model structure. A directed acyclic graph describing the structure of the Bayesian normal two-level varying-intercept and varying-slope regression model used for statistical inference in group-level analyses of all spectral changes, resting-state occipital alpha and motor-related central oscillatory activity, and for the estimation of the signal-to-noise ratio (SNR) on single trials. In the model, *y* represents the outcome variable (the measurements: PSD, power spectral density; difference in PSD—ΔPSD or log-transformed SNR on single trials), ***X*** is the design matrix containing predictor variables coding the type of the artifact reduction method, as well as the condition (EO or EC) in the resting-state (RS) analysis, ***β***_participant_ is the vector of regression coefficients of each participant, ***β*** is the vector of regression coefficients at the group-level, **Σ** is the covariance matrix of the regression coefficients, **σ**_method_ is a vector of standard deviations of the residuals defined for each artifact reduction method, and **Ω** is the correlation matrix of the regression coefficients, modeling the association between regression coefficients. A similar model was used in the within-participants effect size analysis, although the structure of standard deviations was extended by modeling standard deviations separately for each participant and each artifact reduction method, which were pooled from five group-level standard deviations corresponding to the variability of residuals of each method within participants. FC, finger clenching; VEP, visual evoked potentials.

Group-level analyses of all spectral changes, resting-state occipital alpha activity and motor-related central oscillatory activity, were performed with a Bayesian normal two-level regression model with varying-intercept and varying-slope across groups (see Figure 2 and Supplementary Methods 1.1.1). The same model was used to estimate SNR on single trials, where SNR values were log-transformed to conform to normality.

The convergence of Markov chain Monte Carlo (MCMC) algorithm was analyzed by verifying that all estimated parameters had a potential scale reduction statistics ($\hat{R}$) smaller than 1.01, the estimated effective sample sizes in the bulk of distributions and in the tails of distributions were larger than 400 samples (Vehtari et al., 2021). We estimated the uncertainty of our analyses with Monte Carlo standard error (MCSE) (Flegal et al., 2008). To additionally assure the suitability of our models, we visually assessed the quality of convergence *via* posterior parameter trace plots, prior and posterior predictive checks, and we checked that maximum tree depth was not saturated. In order to avoid strong degeneracies inherent to multilevel models and achieve stable convergence (without divergent transitions), we reparametrized all multilevel models to a non-centered parameterization (Betancourt and Girolami, 2013). All models in our analyses were specified and numerically estimated using the probabilistic programming language Stan (Carpenter, 2017).

We used weakly informative prior distributions in all models. Specifically, we used normal prior distributions (μ = 0, σ = 10) for regression parameters and half-Cauchy prior distributions (μ = 0, λ = 2.5) for standard deviations, as recommended by Gelman (2006), for all but the correlation model, where we used a vague normal (μ = 0, σ = 100) prior for the means of the input vectors, a half-Cauchy prior (μ = 0, σ = 2.5) for the standard deviations, and a gamma prior distribution (α = 2, β = 0.1 Juárez and Steel, 2010) for the degrees of freedom parameter.

To estimate the posterior distribution of within-participants effect sizes of the difference between contralateral and ipsilateral VEP responses, we implemented a model similar to that described above and shown in Figure 2. The model was extended by modeling the standard deviation of the residuals using a two-level structure in which we estimated one standard deviation parameter for each participant and each method, pooled from five group-level standard deviations corresponding to the variability of the residuals of each method within participants (Supplementary Methods 1.1.2).

Across-participants effect sizes of the contralateral-ipsilateral difference in VEP responses and SNR on VEP participant-averages were estimated separately for each method. Inference was performed with a single-level normal linear regression (Supplementary Methods 1.1.3).

Correlation analysis of grand-average VEP responses for each of the four stimuli was estimated from the covariance of the bivariate Student's t-distribution, which is more robust to outliers than the normal distribution as assumed when computing Pearson correlation coefficients (Bååth, 2013; Baez-Ortega, 2018) (Supplementary Methods 1.1.4).

## 3. Results

### 3.1. Recovery of eyes-closed occipital alpha activity

**Figure 3:**
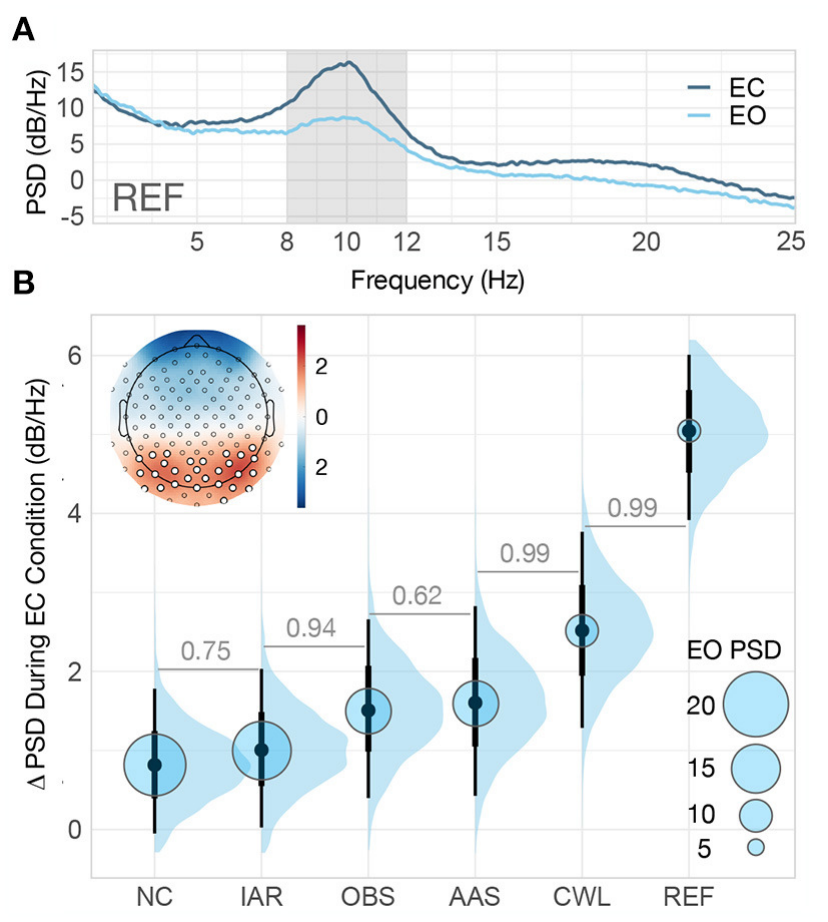
Resting-state spectral analysis. **(A)** Grand-average REF power spectral density (PSD) computed across the entire length of resting-state blocks (0–40 s) for both eyes-open (EO) and eyes-closed (EC) conditions. The shaded region of the spectrum denotes the alpha band (8–12 Hz) on which we computed the mean PSD for the entire resting-state data analysis. **(B)** Posterior distributions of the group-level difference in mean PSD (ΔPSD) in the alpha band between EC and EO conditions for every data variant. The distributions are summarized with the median estimates and 66–95% intervals. The posterior median estimate of the group-level mean PSD in the alpha band during EO condition is coded in the size of the light blue circles. In the upper left corner is the standardized grand-average topography of REF ΔPSD. The summary channel used throughout the resting-state analysis was computed as the mean of all the channels with ΔPSD further than one standard deviation from the mean (highlighted). The numbers between adjacent intervals depict the probability of the method on the right producing a higher ΔPSD than the method on the left, accounting for participants differences. NC, non-corrected signal; IAR, data with the imaging artifact reduced; OBS, data with IAR and reduced BCG artifacts with optimal basis set; AAS, data with IAR and reduced BCG artifacts with average artifact subtraction; CWL, data with IAR and carbon-wire loop artifact correction; REF, data collected outside of the MRI environment.

We first focused on the investigation of the extent to which MR-related artifacts affected the analysis of alpha band EEG activity and the ability of artifact reduction methods to recover the alpha band signal. For that purpose, we compared alpha frequency band power between EO and EC resting-state conditions. We treated the amount of alpha activity associated with the EC condition relative to EO condition in the reference data collected outside the MRI environment (REF) as the benchmark. The topographic distribution of the EC-associated increase in alpha power on REF data (ΔPSD) showed a distinct concentration in the occipital EEG (see Figure 3B upper left corner).

The performance of each method for MR artifact reduction in recovering occipital alpha activity was analyzed with a Bayesian two-level regression model (see Figure 2 and Supplementary Methods 1.1.1). The model included mean PSD values within each block as outcome variables, task condition and data variant as first-level predictor variables, and participants as a second-level grouping variable. To estimate the ability of the methods to recover occipital alpha, we computed the ΔPSD distributions estimated from posterior predictive distributions in the alpha band for each data variant (see Figure 3B).

REF data produced the highest estimates of mean ΔPSD because they were not exposed to the adverse MRI environment. Compared to other MR artifact reduction methods, CWL recovered the largest proportion of alpha power change, however, the change in power was still significantly reduced compared with the mean ΔPSD of the REF data. AAS and OBS methods showed similar results, with AAS yielding a slightly higher estimate of the mean ΔPSD. Both AAS and OBS signals had larger differences in alpha band power than non-BCG corrected (IAR) and NC data (see Figure 3B). Only BCG-corrected data variants supported the finding of a significant change in alpha power between the EO and EC conditions with probability greater than .95, P(ΔPSD > 0) ≥ 0.95.

To gain better insight into the spectral characteristics of each data variant, we examined the estimated PSD levels during the EO blocks, which conveyed information on the baseline PSD level in the alpha band (blue circles in Figure 3B). The lowest level was observed in the REF data and the highest in the NC data. The inverse relationship between baseline PSD level and ΔPSD indicates that the presence of MR artifacts in the alpha band decreases the ability to observe neuronal changes in the alpha band.

### 3.2. Recovery of motor-related spectral activity

**Figure 4:**
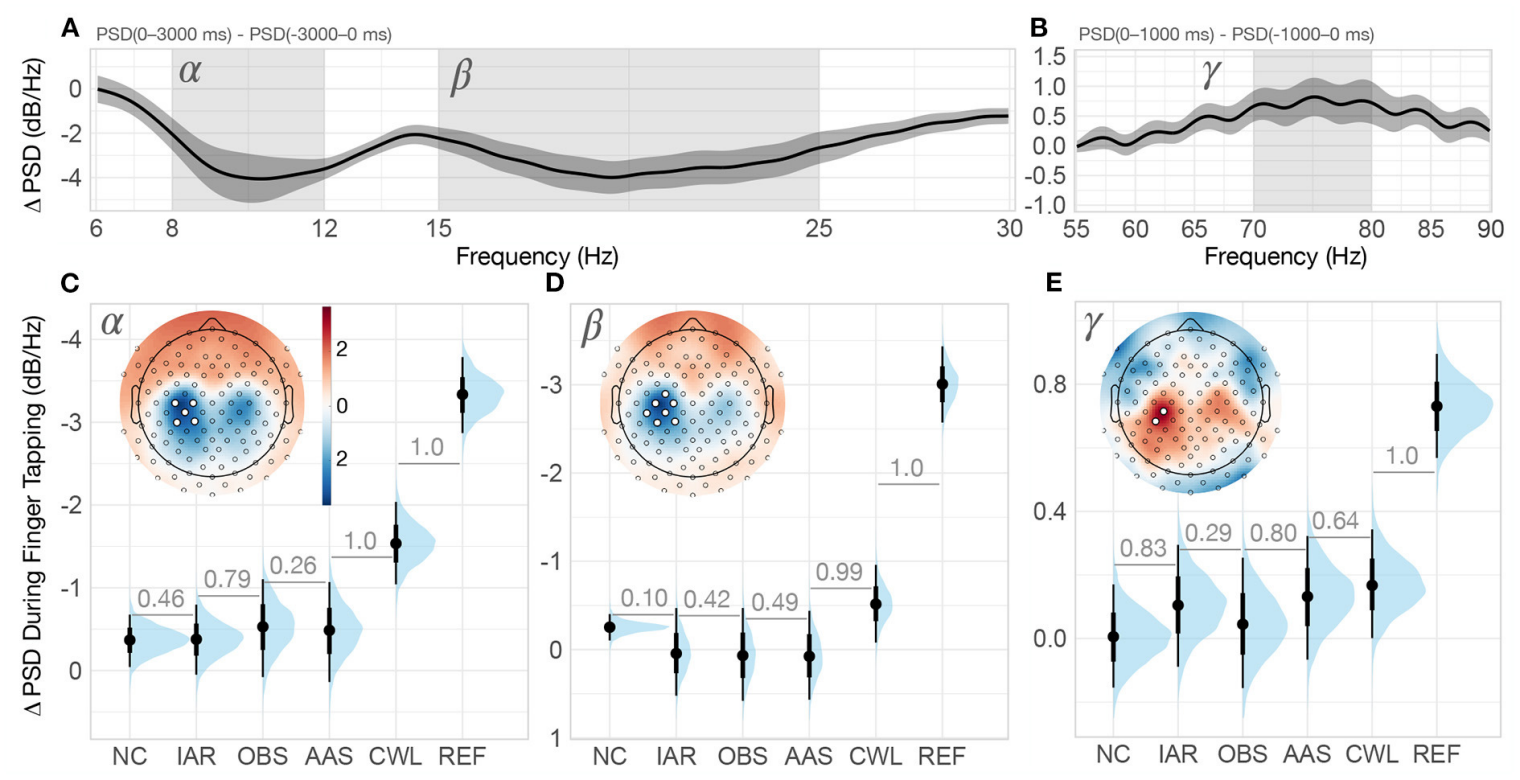
Finger tapping spectral analysis. **(A)** Grand-average REF difference of power spectral density (ΔPSD) during finger tapping period (0–3, 000 ms) relative to baseline (−3, 000–0 ms). The shaded regions of the spectrum denote alpha (8–12 Hz) and beta (15–25 Hz) bands on which we computed the mean ΔPSD for the statistical analysis. The ribbons indicate ±2 between-participants standard errors. **(B)** Grand-average REF ΔPSD at higher frequencies during the finger tapping period (0–1000 ms) relative to baseline (−1000–0 ms). The shaded region of the spectrum denotes the gamma (70–80 Hz) band on which we computed the mean ΔPSD for the statistical analysis. The ribbons indicate ±2 between-participants standard errors. **(C–E)** Posterior distributions of the group-level ΔPSD in the alpha, beta and gamma bands, respectively, for every MR artifact reduction method. The distributions are summarized with the median estimates and 66% and 95% intervals. In the upper left corner of each graph is the standardized grand-average topography of the ΔPSD of the REF data, the color scale in **(C)** applies to topographies in **(D,E)**) as well. The summary channels used for the analysis were computed as the mean of all channels that exceeded two standard deviations in ΔPSD (highlighted with white circles). Numbers between adjacent intervals depict the probability of the method on the right producing a larger ΔPSD than the method on the left, accounting for participant differences. NC, non-corrected signal; IAR, data with the imaging artifact reduced; OBS, data with IAR and reduced BCG artifacts with optimal basis set; AAS, data with IAR and reduced BCG artifacts with average artifact subtraction; CWL, data with IAR and carbon-wire loop artifact correction; REF, data collected outside of the MRI environment.

The finger tapping task allowed us to extend our assessment of the ability of different MR artifact reduction methods to recover EEG spectral content related to event-related (de)synchronization in three different frequency bands—alpha, beta, and gamma—at identified central EEG channel sites (see Methods and Figure 4). The grand-average of ΔPSD between finger tapping and rest showed the expected decreases in the alpha and beta frequency bands and an increase in the gamma frequency band. Task-related (de)synchronizations were greatest in the central area contralateral to the finger tapping hand (left hemisphere), whereas smaller changes were observed on the ipsilateral side.

Again, we used a Bayesian two-level regression model (see Figure 2 and Supplementary Methods 1.1.1) to estimate the extent of task-related (de)synchronization and to compare the results obtained with different MR artifact reduction methods for each frequency band separately. The model included ΔPSD as the outcome variable, data variant as the first-level predictor variable, and participants as the second-level grouping variable.

The posterior distributions of ΔPSD estimates of alpha band activity revealed a considerable desynchronization in REF data that was substantially higher than in any of the MR data variants (see Figure 4C). CWL recovered the largest amount of ΔPSD of all MR data variants and was the only method for which ΔPSD had a higher than 0.95 probability of being lower than 0, P(ΔPSD < 0) ≥ 0.95. AAS, OBS, IAR, and NC yielded similarly weak ΔPSD in the alpha band, with OBS showing slightly better spectral content recovery of the four methods.

The posterior distributions of the beta band desynchronization estimates show a similar pattern to the alpha band. The REF data show a pronounced desynchronization that is substantially higher than for any of the MR data variants (see Figure 4C). Of the MR artifact reduction methods, CWL recovered the largest ΔPSD and is the only method that resulted in a P(ΔPSD < 0) ≥ 0.95. Interestingly, the NC data variant showed a very narrow ΔPSD posterior distribution that also resulted in P(ΔPSD < 0) ≥ 0.95. Weak responses were again observed in AAS, OBS, and IAR data.

Gamma band synchronization analysis showed the weakest effects of finger tapping across the three frequency bands studied. Only REF data resulted in the posterior distribution of ΔPSD estimates that showed a certain greater than zero response, P(ΔPSD > 0) ≥ 0.95 (see Figure 4E). CWL, AAS, and IAR appeared to recover a similar amount of gamma band synchronization, while OBS fared slightly worse.

### 3.3. Recovery of visual evoked potentials

Lastly, to provide a comprehensive evaluation of MR artifact correction methods in both the frequency and time domains, we extended our evaluation to the analysis of visual evoked potentials (VEP) resulting from checkerboard pattern stimulation of the four quadrants of the visual field. VEP responses consist of prominent peaks following stimulus onset. Responses to different stimuli can differ in peak latency, amplitude, or polarity (Luck, 2014), based on the specific properties of the stimuli. Because ERP studies often investigate differences in ERP components due to experimental conditions, we focused on the ability to distinguish between responses to ipsilateral and contralateral visual stimuli.

**Figure 5:**
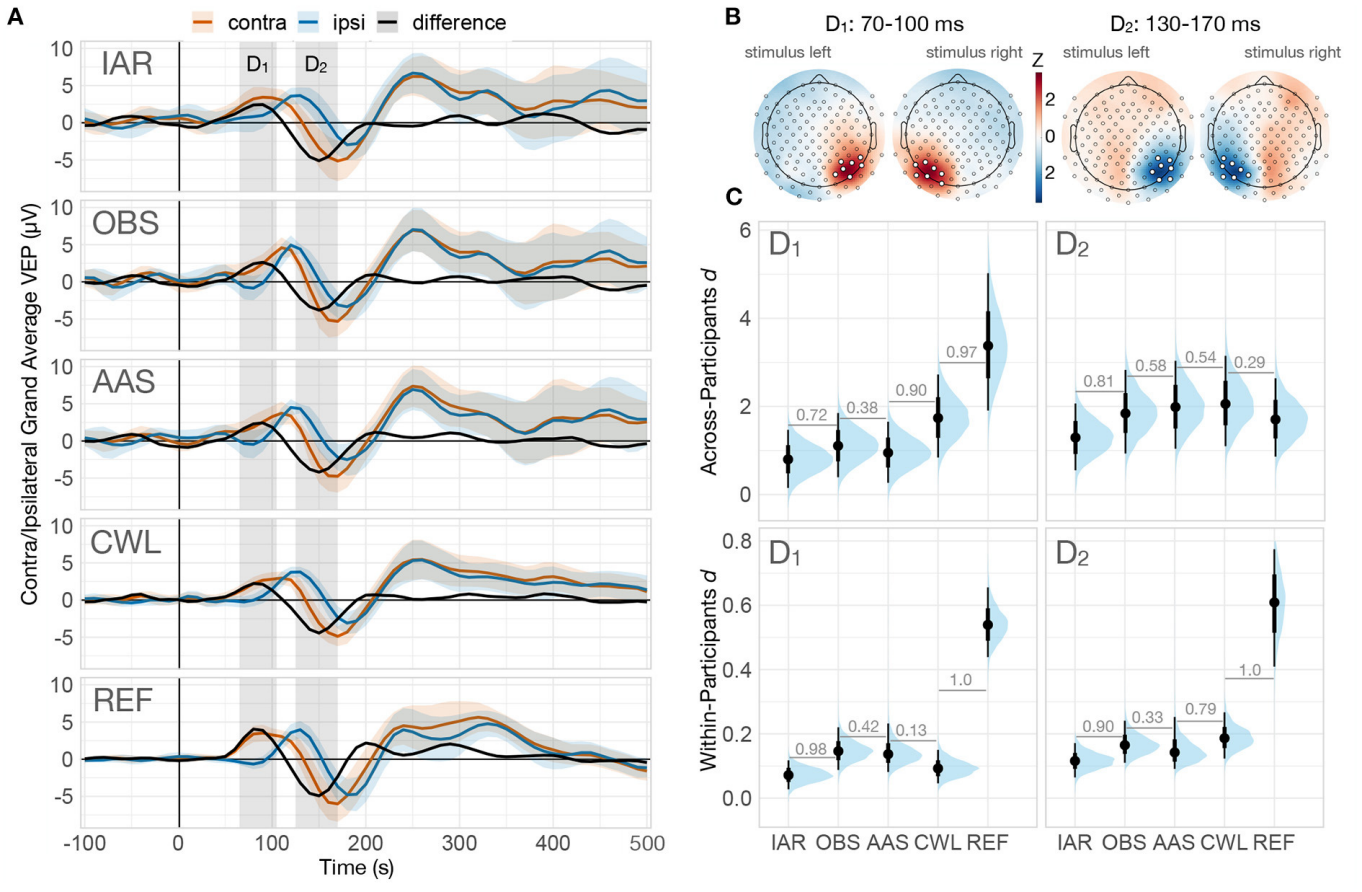
Effect size analysis of contralateral-ipsilateral VEP difference. **(A)** Grand-average VEPs computed on corresponding summary channels for contralateral and ipsilateral trials, along with the difference wave (contralateral—ipsilateral) of each data variant. The shaded regions denote the two prominent peaks of the difference wave (D_1_: 70–100 ms, D_2_: 130–170 ms). The ribbons indicate ±2 across-participant standard errors (omitted on the difference wave for clarity). **(B)** Standardized grand-average topographies of both difference wave peaks and stimulus hemifields. The summary channels used for the analysis were computed as the mean of all channels that exceeded two standard deviations from the mean (highlighted). **(C)** Posterior distributions of the across-participants effect size of both peaks (top row) and the posterior distribution of the group-average of within-participants effect sizes (bottom row). Numbers between adjacent intervals depict the probability of the data variant on the right producing a larger effect size than the one on the left, controlling for participant variability. IAR, data with the imaging artifact reduced; OBS,data with IAR and reduced BCG artifacts with optimal basis set; AAS, data with IAR and reduced BCG artifacts with average artifact subtraction; CWL, data with IAR and carbon-wire loop artifact correction; REF, data collected outside of the MRI environment.

As a first step of the analysis, we reviewed the grand-average contralateral and ipsilateral VEP responses and their difference for each data variant evaluated (see Figure 5A). We observed two peaks in the difference wave in each data variant, D_1_ and D_2_. A review of the topographies in the corresponding time windows revealed peak responses in occipito-parietal channels contralateral to the presented stimulus (see Figure 5B).

We then assessed the performance of artifact reduction methods from two different perspectives of ERP analyses: (i) the ability to identify ERP differences across trials within a participant (single participant analysis) and (ii) the ability to identify differences across a group of participants (second-level group analysis). To this end, rather than examining absolute differences in μ*V*, we focused our analysis on estimating effect sizes obtained in different data variants. For within-participant effect sizes, we investigated average effect sizes computed on differences across trials for each participant independently. The analysis was performed using a two-level Bayesian linear regression model (Supplementary Methods 1.1.2), with single-trial amplitude as the outcome variable, stimulus side (ipsilateral vs. contralateral), and data variant as first-level predictor variables, and participants as the second-level grouping variable. For effect sizes across participants, we computed effect sizes for the differences between participants' VEP averages and analyzed them using a single-level Bayesian linear regression model (Supplementary Methods 1.1.3) with mean amplitude difference as the outcome variable and data variant as the predictor variable.

Analysis of within-participant effect sizes revealed medium effect sizes in the REF data and small effect sizes in all MR data variants for both D_1_ and D_2_ mean amplitude differences (see Figure 5C, bottom row). The performance of the MR reduction methods was similar, with only IAR resulting in the smallest effect sizes. Analysis of across-participant effect sizes showed large effect sizes for all data variants with a different pattern for the two difference peaks (see Figure 5C, top row). For D_1_, the observed effect size was significantly higher in the REF data than in MR data variants, with the CWL data variant having a robustly higher effect size than other MR variants. For D_2_, effect sizes were surprisingly similar across variants, with the smallest effect size observed only in the IAR MR data variant.

**Figure 6:**
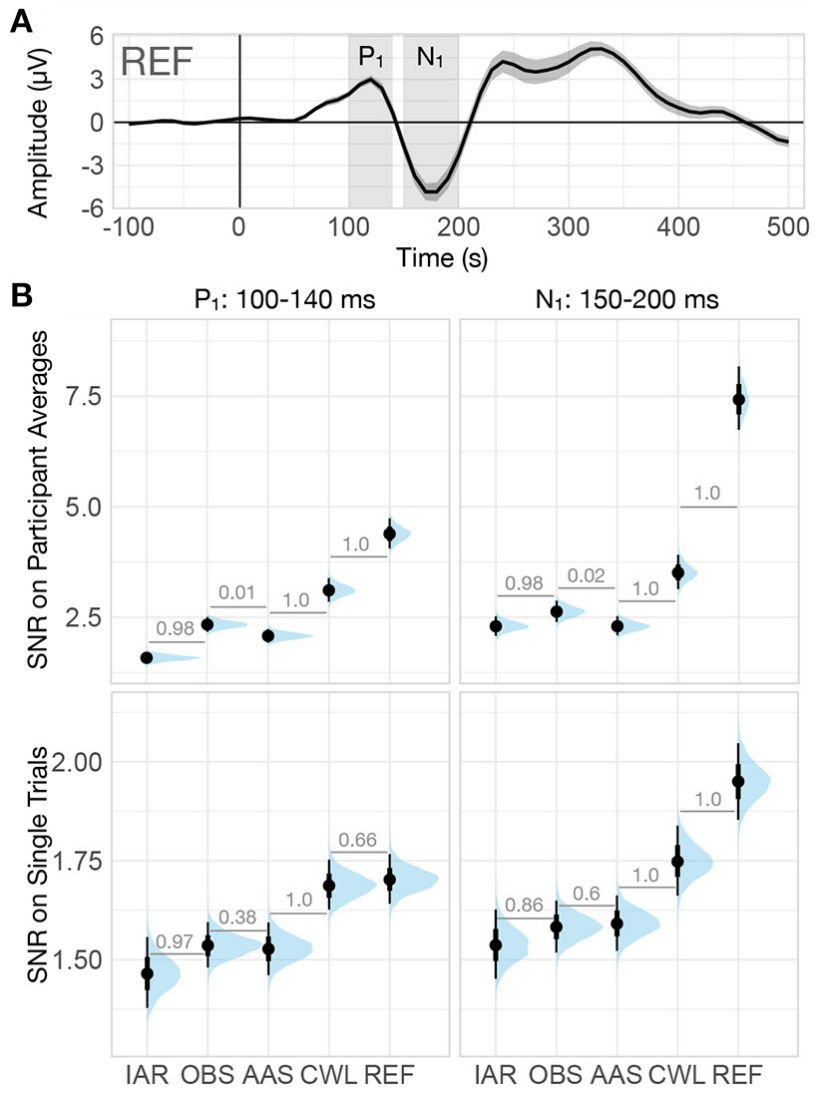
Signal-to-noise ratio (SNR) of VEP. **(A)** Grand-average VEP computed on the summary channel of data collected outside of the MRI environment (REF). The shaded regions denote the two prominent peaks of the VEP (P_1_: 100–140 ms, N_1_: 150–200 ms). The ribbons indicate ±2 between-participants standard errors. **(B)** Posterior distributions of SNR of both VEP peaks computed on participant averages (top row) and on single trials (bottom row) for every data variant. The distributions are summarized with median estimates and 66–95% intervals. Numbers between adjacent intervals depict the probability of the method on the right producing a larger SNR than the method on the left, accounting for participant differences. IAR, data with the imaging artifact reduced; OBS, data with IAR and reduced BCG artifacts with optimal basis set; AAS, data with IAR and reduced BCG artifacts with average artifact subtraction; CWL, data with IAR and carbon-wire loop artifact correction; REF, data collected outside of the MRI environment.

Since the presence of noise is a key determinant of the ability to identify the effects of experimental variations on ERP peaks and components, we also investigated the signal-to-noise ratio (SNR) observed in different data variants. We examined SNR at two levels: (i) the SNR_t_ observed across individual trials, and (ii) the SNR_a_ observed on average ERPs (see Section 2.5.2) for the two most prominent peaks, P_1_ and N_1_, within 100–140 ms and 150–200 ms time windows, respectively (see Figure 6A).

Similarly to the effect size analysis, for the trial-level SNR analysis, we used a two-level Bayesian linear regression model with SNR_t_ for each trial as the outcome variable, data variant as the first-level predictor variable, and participant as the second-level grouping variable. We modeled the residuals separately for each method (see Figure 2). For the analysis of the average ERP SNR, we used a single-level Bayesian linear regression model with SNR_a_ as the outcome variable and data variant as the predictor variable.

The analysis revealed significant variability in SNR between data variants. While SNR was markedly higher at the average ERP level than at the single-trial level, for P_1_ and N_1_ both SNR estimates were highest for REF data (see Figure 6B). Though with SNR considerably smaller than for REF data, CWL performed substantially better than other MR reduction methods at both single trial level and the average ERP level. At single-trial level, both AAS and OBS performed similarly and substantially better than IAR, while at the average ERP level, OBS yielded a significantly higher SNR than AAS.

**Figure 7:**
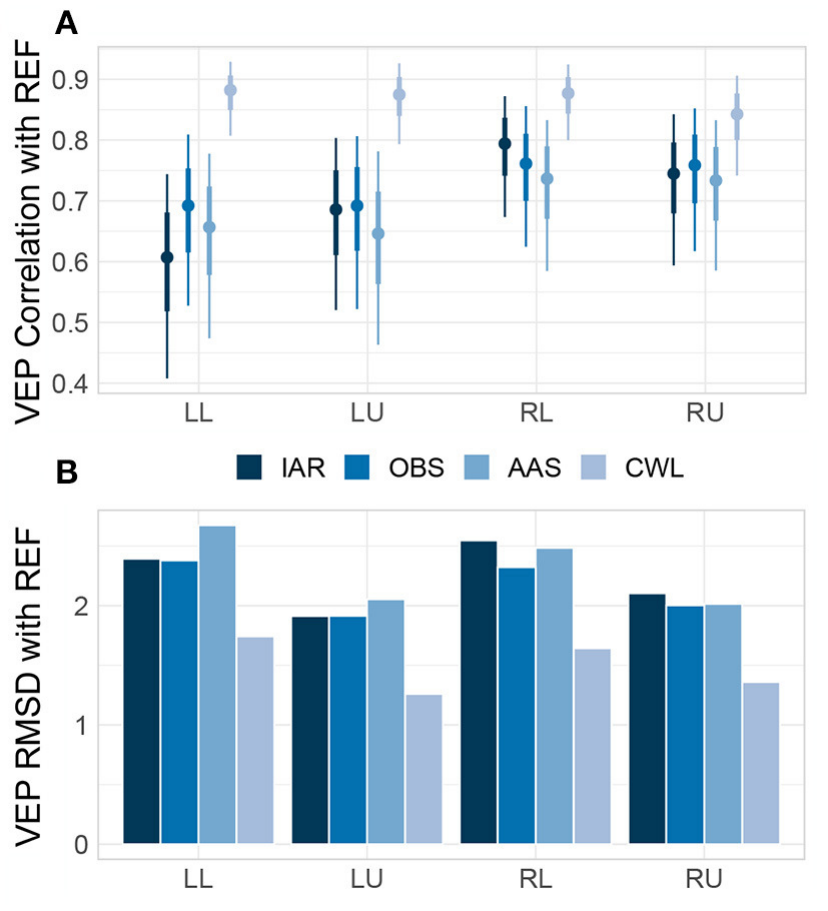
Grand-average VEP correlations and RMSD. Posterior distributions of correlation coefficients **(A)** and root-mean-square deviations (RMSD) **(B)** between the grand-average VEP of each MR data variant and the grand-average VEP of data collected outside of the MRI environment (REF) for each type of visual stimulus. Correlation distributions are represented with median estimates and 66–95% intervals. LL, left lower; LU, left upper; RL, right lower; RU, right upper; IAR, data with the imaging artifact reduced; OBS, data with IAR and reduced BCG artifacts with optimal basis set; AAS, data with IAR and reduced BCG artifacts with average artifact subtraction; CWL, data with IAR and carbon-wire loop artifact correction.

Lastly, we checked how the morphology of the grand-average VEP of all MR data variants compared to the morphology of the VEP of REF data. We estimated similarity using robust correlations between the time series and dissimilarity using the RMSD between the time series, both separately for each type of stimuli. Across the grand-average VEPs, the CWL data variant robustly showed the highest correlations (see Figure 7A) and the smallest RMDS (Figure 7B) from REF VEPs, whereas other methods performed comparably.

## 4. Discussion

The aim of this study was to evaluate and compare the performance of the most widely used and readily available MRI artifact reduction methods in order to provide empirically supported guidelines for the design of new EEG-fMRI studies and for the selection of processing options that ensure optimal analysis of EEG data acquired simultaneously with fMRI. To this end, we collected and analyzed data using three different paradigms representative of continuous EEG, task-related (de)synchronization, and ERP studies. We compared results obtained outside the MR environment with those obtained using simultaneous EEG-fMRI recording preprocessed with different MR artifact reduction methods.

### 4.1. MR artifact reduction performance in the frequency domain

Resting-state data with eyes-opened and eyes-closed conditions allowed us to investigate the ability to identify changes in frequency spectra in the continuous EEG signal. Previous studies have reported robust increases in occipital alpha band power during the EC condition (Kirschfeld, 2005) so this paradigm seemed particularly well suited for evaluating the performance of each method in reducing the BCG artifact as it overlaps with alpha activity in the frequency domain (see Figure 1D).

The evaluation revealed a marked reduction in the ability to observe alpha band power reduction in the continuous EEG signal acquired during simultaneous EEG-fMRI data acquisition. IAR alone did not effectively recover spectral content in the alpha band, supporting the necessity of additional processing to reduce BCG artifact previously shown by van der Meer et al. (2016), Liu et al. (2012), and Lin et al. (2022). While Grouiller et al. (2007) reported that BCG correction did not improve spectral content in the alpha band and above, our data show that BCG artifact reduction using either OBS or AAS substantially improves the ability to recover alpha band power change compared to IAR alone. Our evaluation, similar to that of Wang et al. (2018), showed similar performance of OBS and AAS methods. As previously reported by van der Meer et al. (2016), our evaluation replicated significant further improvement in alpha band spectral content recovery enabled by CWL.

Next, we selected the finger tapping paradigm to investigate event-related (de)synchronization. We chose finger tapping because it has previously been reported to result in robust motor action-related desynchronization in the alpha and beta bands and synchronization in the gamma band on pericentral EEG sites (Ritter et al., 2009). Specifically, both alpha and beta band activity tend to increase during the idle condition of the motor cortex, as a result of synchronization of neurons in the primary motor cortex (Ritter et al., 2009), a similar phenomenon to occipital alpha activity observed in EC conditions. In contrast, motor-related gamma activity tends to increase during the onset of movement (Ball et al., 2008; Darvas et al., 2010). The broader spectral nature of motor-related oscillations therefore allowed us to investigate beta and gamma band activity in addition to alpha activity. The inclusion of a motor task also allowed us to study how well MR artifacts reduction methods perform at other sites on the scalp. We were specifically interested in the ability of MR artifact reduction methods to recover gamma band oscillations, as previous evaluation studies focused primarily on robust occipital alpha oscillations (e.g., Liu et al., 2012; Hermans et al., 2016; van der Meer et al., 2016), whereas recovery of motor-related oscillations in the gamma band has previously been reported only by Kim et al. (2015).

As in the resting-state analysis, MR artifacts markedly impacted the ability to observe task-related (de)synchronizations in all frequency bands. Surprisingly, in contrast to the resting-state analysis, no MR artifact reduction method other than CWL substantially improved the results in the alpha or beta bands. This result was particularly interesting because a number of studies (e.g., Niazy et al., 2005; Debener et al., 2008; Mullinger et al., 2013) reported weaker BCG and consequently lower residual artifacts on central compared with occipital EEG sites, which led us to expect better performance in motor-related activity recovery compared with occipital resting-state alpha.

One possible cause for reduced performance of BCG artifact correction methods could be increased vibrations due to finger tapping, which may have propagated from the hand to the head and induced additional motion-related artifacts. To test this hypothesis, we estimated the ΔPSD in the studied frequency bands on the IAR signal of the four CWLs attached to the participants' heads. These were isolated from the scalp, so their signals contained only MR-induced potentials. As movements due to finger tapping were not present in the baseline period, a positive ΔPSD in the alpha and beta bands would imply a presence of a motion-induced signal that may have counteracted event-related desynchronization in the alpha and beta frequency bands. This possibility was confirmed by the supplemental analysis, which revealed a presence of significant increase in both alpha and beta band power during finger tapping (see Supplementary Analyzes 2.1 for details). The slightly better performance of OBS over AAS may be due to the higher sensitivity of AAS to random motion. Namely, changes in the shape of the induced artifacts violate the fundamental assumption of AAS, that the artifacts considered for template computation do not change over time. OBS, on the other hand, estimates the artifact shape by its principal components (Niazy et al., 2005), which may make it more resilient to temporal changes in the induced artifacts.

An additional curiosity was the observation of a higher and more robust beta band ΔPSD in NC compared to IAR, OBS, and AAS data. One possibility could be that finger tapping induced movements resulted in altered imaging artifacts with a slightly different spectral response than the imaging artifacts during baseline, leading to suboptimal attenuation of the beta band signal during finger tapping. However, this possibility should be explored further.

In comparison to the alpha and beta bands, MR artifact reduction methods did somewhat improve relative sensitivity to event-related gamma band synchronization. Of the MR reduction methods, OBS appeared to perform the worst and even decreased the information recovered by IAR, suggesting a possible loss of neuronal information at higher frequencies. This possibility was suggested by Liu et al. (2012), proposing that the removal of the first few principal components may lead to overcorrection of the data. This could possibly be mitigated by including fewer principal components in the template computation, however, this option would need further investigation. The similar performance of the IAR, AAS, and CWL methods might imply that neither the AAS method nor the CWL signals captured additional artifacts in the 70–80 Hz band. Importantly, the CWL data variant was the only MR data variant that revealed task-related gamma synchronization with a probability greater than 0.95.

Due to the popularity of the FASTR algorithm for IAR and its availability in the fMRIb EEGLAB plug-in (Niazy et al., 2005), we also evaluated the use of FASTR as the initial preprocessing step before CWL regression. With this supplementary analysis we wanted to assess whether a combination of FASTR and CWL provides additional benefits over the use of basic AAS prior to CWL, which was included in the main evaluation. The recovery of spectral content of both approaches to IAR performed prior to CWL regression was comparable, thus the choice between the two algorithms for IAR does not play a critical role in spectral EEG analyses (see Supplementary Figure S7).

### 4.2. MR artifact reduction performance in the time domain

Analysis of VEPs allowed us to evaluate the performance of MR artifact reduction methods in ERP studies. In particular, we focused on evaluating the ability to identify task- or stimulus-related components and component differences. For this reason, we used different visual stimuli and analyzed effect sizes for differences in ipsilateral vs. contralateral visual stimulation and ERP SNR. Furthermore, we investigated both across-trial, within-participant measures and measures computed across average participant ERP responses.

Evaluation of effect sizes obtained across participants allows us to estimate the ability to identify group-level task- or stimulus-related differences in ERP responses. Investigation of the first difference wave peak, D_1_, showed a clear advantage of REF over all MR data variants, followed by CWL, which outperformed AAS, OBS, and IAR. These findings reflect both a stronger response to the contralateral stimulus presentation as well as smaller variability in the REF data (see Figure 5A). While the CWL data variant showed a similar attenuation of the response to contralateral stimuli, the higher effect size can be attributed to smaller VEP variability. Surprisingly, no practical differences between the data variants, including REF data, were observed in D_2_, with the exception of a somewhat reduced effect size in IAR. This pattern or results might suggest an actual difference in VEP response in the two environments due to lightning and stimulus display conditions.

Evaluation of within-participant effect sizes enabled us to estimate the ability to identify task- or stimulus-related differences in ERP responses at the individual level. These results were more consistent with prior expectations of the superiority of the REF data, as they yielded markedly higher effect sizes than the MR data variants in both time windows of interest. As expected, IAR performed worst in both time windows, surprisingly though, OBS and AAS outperformed CWL in time window D_1_. Visual inspection of the magnitudes of the differences and their variability in both time windows for all participants (see Supplementary Figure S5) revealed somewhat higher variability in difference estimates within participants and less variability across participants in CWL compared to both ASS and OBS data variants, consistent with lower effect sizes within participant and higher across participants. One explanation for this conflicting result might be that some artifact peaks in the CWL data were strongly attenuated during VEP averaging of the trials, resulting in higher across-participants effect size, whereas in OBS and AAS, the residuals might have been more time-locked, allowing them to bypass the desired attenuation from averaging. Review of the individual data prompted us to investigate to what extent individual differences in D_1_ and D_2_ were correlated across methods. The results (see Supplementary Figure S6) indicated overall low correlations of REF results with all MR data variants for D_1_ and low to moderate (CWL) and high (OBS) in D_2_. Overall, the observed within-participant effect sizes suggest that the ability to detect ERP differences in a single participant is significantly reduced in EEG data acquired simultaneously with fMRI, necessitating a larger number of trials.

Evaluation of trial-level SNR_t_ enables us to estimate the sensitivity to ERP signals at the single-trial level, whereas SNR_a_ reflects the ability to discern ERP peaks in the average participant ERP time series. As expected MR artifacts significantly reduce SNR at both single-trial level and the average ERP level, while trial averaging significantly increases SNR. Further, CWL substantially outperforms all other MR reduction methods at both single-trial level and trial-average levels. OBS, in turn, showed better SNR performance on participant averages than AAS, despite both methods retaining similar SNR on single trials. This indicates that additional averaging of trials might have led to attenuation of non-time-locked residual artifact peaks. Previous studies reported conflicting results from SNR analyses on OBS and AAS data, with some reporting better SNR for OBS (e.g., Krishnaswamy et al., 2016; Marino et al., 2018) and others for AAS (e.g., Liu et al., 2012; Wang et al., 2018). Shams et al. (2015) reported higher SNR for OBS than for AAS and suggested that individual tuning of the number of principal components used in OBS may improve SNR when using this method.

Jointly the evaluation of MR artifact reduction methods in the time domain suggests that CWL regression might have led to a slightly higher degree of ERP peak attenuation when compared with OBS and AAS. However, the noise due to MR artifacts was significantly lower, both on single trials and on participants' averages. This resulted in superior SNR at both levels of analysis and might have also led to more consistent results across participants, which in turn led to higher effect sizes at the group level. The better noise reduction provided by CWL could also underly a substantive advantage of CWL over other MR artifact reduction methods in recapturing the ERP morphology, as revealed in the analysis of similarities and differences with the REF ERP time courses. Correlation and RMSD analyses also showed a slight advantage of OBS over AAS and IAR, which suggested, in addition to marginally better SNR, that OBS does leave lower artifact residuals in the signals than AAS (as reported by Niazy et al., 2005 and Marino et al., 2018).

An important consideration of evaluation in the time domain is the time locking of events with the MR signal. In concurrent EEG-fMRI recording, the onset of events is frequently locked to the onset of BOLD volume acquisition. Any residual MR imaging artifacts are therefore also time-locked to the observed ERP, which could lead to enhancement rather than attenuation of residuals during averaging and to systematic deformations of ERPs acquired in simultaneous EEG-fMRI recording. This might have led to reduced correspondence with REF ERP time courses for non-CWL methods. A possible approach to reducing such time-locked artifact could be the addition of slight jittering of event onsets. Jittering across the range of about 50 ms should counteract the MR imaging artifact time-locking without affecting the accuracy of the fMRI analyses due to the slow evolution of the BOLD signal. The impact of this strategy on the effect of IAR, OBS, and AAS results should be investigated in further studies.

The a posteriori analysis of the performance of FASTR and AAS algorithms for IAR prior to CWL yielded similar results in recovering visual evoked potentials as in recovering spectral content, where the performance of both methods was comparable and the choice between the two algorithms should not significantly affect the quality of EEG data in ERP research (see Supplementary Figure S8).

### 4.3. Significance and limitations

To address the aims of the study, we kept the complexity of our evaluation within reasonable limits. We achieved this by focusing only on readily available MR artifact reduction methods that were easy to access and limiting all methods to their default parameters to simulate the situation relevant for a majority of researchers. If these restrictions had been relaxed, we would have strayed from the central thread of our study, which was to evaluate methods that an everyday researcher would have at hand and ready to use. However, by limiting methods to their default parameters, we were unable to explore performance enhancements possibly afforded by parameter optimization. Nevertheless, our findings should provide valuable practical information and possibly motivate and provide directions for future evaluation studies.

In every evaluation analysis performed in this study, we treated the REF signal, collected outside the MRI environment, as the benchmark to which we compared differently preprocessed variants of data collected simultaneously with fMRI. Thus, the evaluation was based on the assumption that the observed neuronal responses were the same or very similar in the two environments. The most obvious factors that could have led to a violation of this assumption include (i) the effects of body position on brain activity, e.g. increased cortical inhibition in the supine position (Spironelli et al., 2016), as the participants were seated during REF data acquisition and in a supine position in the scanner during fMRI acquisition, (ii) the presence of loud acoustic noises and different lighting conditions in the scanner, and (iii) the decline in attention and vigilance as the participants performed all three behavioral tasks first outside the MRI environment and then again in the MRI.

The fact that each task had to be performed twice limited the number of tasks we were able to include in the study. With that in mind, we selected and designed tasks that enabled us to evaluate the performance of a relatively wide array of different neuronal responses from EEG data. However, there are countless additional ways in which method performance could be analyzed, ranging from various ERP to source-space EEG functional connectivity analyses. In addition, further improvements in artifact reduction could be enabled by auxiliary processes that support effective artifact correction, such as accurate R-peak detection in ECG signals, that we did not explore.

It needs to be noted that VEP analyses were conducted on data of summary channels, which were computed from different numbers of channels in different evaluation analyses (8 channels per hemisphere in the effect size analysis and 30 channels in the SNR, correlation, and RMSD analyses). This could lead to different degrees of attenuation of non-time-locked potentials, which may include both artifacts and non-event-related neuronal activity. The attenuation factors were $\sqrt{8}\approx 2.8$ and $\sqrt{30}\approx 5.5$ in the effect size case and in all other VEP analyses, respectively. This should be taken into account when relating results from these analyses.

An important consideration is the specific MR environment in which the data were collected, as the evaluated methods may perform differently depending on the characteristics of the MR-related artifacts. These differences can stem from (i) the specific sequences used, which would impact the shape and frequency structure of the MR imaging artifacts, (ii) the strength of the magnetic field, which would impact the amplitude of the BCG and motion-related artifacts, and (iii) other vendor and model-specific characteristics, such as the extent of vibration caused by the helium pump. Our study was based on data collected using a multiband BOLD sequence on a 3 T Philips scanner, however, different results might be obtained in a different environment. Taking that in consideration, we feel that our results should be representative of modern fMRI scanning environments. It is also reasonable to expect that a method that performs poorly at 3 T will perform even worse at higher magnetic fields since any artifact should be potentiated in a stronger magnetic field.

Further, in our evaluation we initially included only one method for IAR. Including additional IAR methods in the evaluation would significantly expand the number of possible combinations of IAR and BCG artifact removal methods, which we wanted to avoid. As imaging artifacts are deterministic and different methods use the same or very similar implementations, we presumed that the results of IAR would not change substantially, which was later supported by the a posteriori analysis where we evaluated the performance of FASTR for IAR in combination with CWL. Employing a single, relatively conservative IAR method for all the main analyses allowed us to focus on the evaluation of methods for the removal of more complex and unpredictable BCG and motion-related MR artifacts.

Lastly, the addition of the CWL system in our evaluation revealed the inability of conventional methods, AAS and OBS, to recover motor-related alpha and beta activity. Without the results of the CWL-processed datasets, we would not have observed any spectral activity in the alpha or beta band and would have considered the possibility that this oscillatory activity was not expressed to the same extent in the MRI as it was outside the MRI environment. This finding highlights the importance of using a reference signal system in studies involving voluntary motor actions, as it allows independent acquisition of motion-related artifactual signals and their removal to the extent that is not possible with other methods. We therefore advise that tasks requiring any voluntary motor responses, however weak they may be, utilize a reference signal-based approach to artifact correction to ensure high-quality EEG data for further analyses.

### 4.4. Guidelines for effective reduction of MR artifacts

As indicated in the previous sections, selecting the most appropriate method for artifact reduction depends to some extent on the nature of the study and the analyses to be conducted. Both previous studies and our empirical results suggest that reference signal methods outperform conventional methods while allowing real-time data processing. The major drawback of many reference signal methods is the added hardware complexity, although the CWL system does not require extensive modifications or additional time for cap preparation.

***Minimizing exposure to electromagnetic induction*** is the first step in achieving high-quality EEG data collected simultaneously with fMRI. A few straightforward preventive measures include (i) reducing movements of the leads connecting the EEG cap to the amplifiers by fixing them to the floor of the scanner bore with self-adhesive tape and weighting them with sand bags (Allen et al., 1998), (ii) fixing participant's head with cushioning to reduce movements (Bénar et al., 2003), (iii) placing the amplifiers inside the scanner bore (if warranted by the manufacturer), as shorter connections to the cap make the system less susceptible to MR artifacts (Assecondi et al., 2016), and (iv) turning off the cryostat helium pump and the ventilation system if possible and when higher frequency content is of interest.

***Proper data acquisition*** will allow effective artifact correction in post-processing. The amplitudes of the electrical currents induced during BOLD imaging can easily exceed the measurement range of certain EEG amplifiers, so it is important to carefully select the most appropriate acquisition gain and high-pass and low-pass filter cutoff frequencies in order to avoid signal saturation (Allen et al., 2000). The precise temporal synchronization between the EEG and MRI clocks is a critical factor for effective IAR, which requires precise markers indicating the onset of MRI slice/volume acquisition. Likewise, the effectiveness of AAS and OBS is directly dependent on the precision and accuracy of R-peak detection, therefore a high-quality ECG signal is essential. In cases where clock synchronization can not be established, it is essential to sample EEG data at the highest possible sampling frequency (≥5 kHz) to allow better temporal alignment of artifact sections in post-processing (Allen et al., 2000).

***Imaging artifact reduction*** usually represents the first step in MR artifact reduction. The deterministic nature of the artifact and the fact that most of the contemporary studies utilize MR-EEG clock synchronization make the selection of an algorithm for IAR easier than for BCG artifact reduction. In general, AAS leads to more conservative IAR, whereas FASTR can lead to more intense IAR, possibly attenuating neuronal information in addition to some BCG artifacts (Bullock et al., 2021).

***BCG and motion-related artifact correction*** should be performed after IAR. Based on our evaluation results, we advise investing in a CWL system, particularly for studies that can not reduce movements in the scanner (e.g., tasks requiring motor action, pediatric and clinical populations) or when higher-frequency EEG content is of interest and the helium pump or ventilation systems cannot be turned off. In cases where reference signals were not collected and the signals of interest are affected by the helium pump artifacts, we advise researchers to utilize either the sliding-window AAS approach proposed by Rothlübbers et al. (2015) or rsPCA proposed by Kim et al. (2015). For conventional BCG artifact reduction, we propose a less rigorous AAS when investigating weaker spectral effects at higher frequencies and OBS for ERP studies because it seemed to yield a higher SNR. In our evaluation, we observed only marginal differences between the efficacy of AAS and OBS, which is in line with conflicting conclusions regarding AAS and OBS in the literature. We did use both methods with default parameters, and as suggested by Shams et al. (2015), further improvements may be achieved with additional parameter optimization. Motion-related artifacts can be very delicate to reduce without a reference-signal method, and even when such methods are used, motion should be minimized. Using head restraints is therefore the most straightforward approach to reducing motion artifacts (Bénar et al., 2003). An additional post-processing approach to motion-related and muscle artifacts reduction using ICA was proposed by Mayeli et al. (2016). Although we have not evaluated ICA for motion-artifact reduction, we suggest researchers experiment with ICA for additional artifact reduction, especially when other forms, such as CWL, are not available.

## 5. Conclusion

In conclusion, we evaluated MR artifact reduction methods on three different tasks eliciting diverse neuronal responses measured by EEG to gain deeper insights into how two conventional and readily available methods, AAS and OBS, along a more advanced reference signal method, CWL, perform in recovering distinct neuronal responses. Our results indicate that CWL provides superior performance in both continuous and event-related spectral analyses across frequency bands, especially when tasks require motor actions that introduce additional artifacts. In the time domain, CWL yields stronger group-level effect sizes, superior single-trial and average ERP SNR, and ERP time series recovery. For these reasons, we advise investing in a CWL system for simultaneous EEG-fMRI recordings. Inferior to CWL, AAS and OBS yield comparable results, wherein OBS appears to perform more rigorous artifact reduction, resulting in better performance in the time domain but some signal loss in high-frequency spectral content. OBS could benefit from additional parameter optimization to individual study and data characteristics.

## Data availability statement

The raw data supporting the conclusions of this article will be made available by the authors, without undue reservation. The processed and exported datasets and data analysis scripts used in this study can be found in Open Science Framework online repository: https://osf.io/2ey74/.

## Ethics statement

The studies involving human participants were reviewed and approved by Ethics Committee of the Faculty of Arts, University of Ljubljana, Slovenia. The patients/participants provided their written informed consent to participate in this study.

## Author contributions

AK, AM, and GR: conceptualization and methodology. AK, AM, JD, and GR: software. AK: formal analysis, writing–original draft, visualization, and project administration. AK, AM, and NP: investigation. AK, AM, NP, JD, and GR: writing–review and editing. GR: supervision and funding acquisition. All authors contributed to the article and approved the submitted version.

## Funding

This research was supported by the Slovenian Research Agency's grants J3-9264, J7-8275, P3-0338, and P5-0110.

## Conflict of interest

GR consults for and holds equity in Neumora Therapeutics and Manifest Technologies. JD consults for Manifest Technologies. JD, AK, and AM have previously consulted for Neumora (formerly BlackThorn Therapeutics).

The remaining author declares that the research was conducted in the absence of any commercial or financial relationships that could be construed as a potential conflict of interest.

## Publisher's note

All claims expressed in this article are solely those of the authors and do not necessarily represent those of their affiliated organizations, or those of the publisher, the editors and the reviewers. Any product that may be evaluated in this article, or claim that may be made by its manufacturer, is not guaranteed or endorsed by the publisher.
