# Supplementary material for: Evaluation and comparison of most prevalent artifact reduction methods for EEG acquired simultaneously with fMRI
Source: Front Neuroimaging. 2022 Aug 29;1:968363. doi: 10.3389/fnimg.2022.968363 (PMC10406266; doi:10.3389/fnimg.2022.968363)
Supplement: Supplementary file 1 [file Data_Sheet_1.PDF]

frontiers

Supplementary Material

1 SUPPLEMENTARY METHODS

1.1 Statistical Inference

1.1.1 Spectral Content Analyses (RS and Finger Tapping) and SNR on Single Trials

The group-level analyses of all spectral changes, resting-state occipital alpha and motor-related central oscillatory activity, and for the estimation of the SNR on single trials were performed with a Bayesian normal two-level regression model with varying-intercept and varying-slope across groups S1. We reparametrized the model to noncentered parametrization to achieve stable convergence. The model was specified and numerically estimated using the probabilistic programming language Stan (Team, 2022).

$$
\begin{aligned}
y_{\mathrm{i}} &\sim \mathrm{Normal}(X_{\mathrm{i}}\boldsymbol{\beta}_{\text{participant[i]}}, \boldsymbol{\sigma}_{\text{method[i]}}) \\
\boldsymbol{\beta}_{\text{participant}} &\sim \mathrm{Normal}(\boldsymbol{\beta}, \boldsymbol{\Sigma}) \\
\boldsymbol{\beta} &\sim \mathrm{Normal}(0, 10) \\
\boldsymbol{\Sigma} &= \mathrm{var}(\boldsymbol{\beta})\ \boldsymbol{\Omega}\ \mathrm{var}(\boldsymbol{\beta}) \\
\mathrm{var}(\boldsymbol{\beta}) &\sim \text{Half-Cauchy}(0, 2.5) \\
\boldsymbol{\Omega} &\sim \mathrm{LKJcorr}(2) \\
\boldsymbol{\sigma}_{\text{method}} &\sim \text{Half-Cauchy}(0, 2.5),
\end{aligned}
\tag{S1}
$$

where y is the outcome variable, $\boldsymbol{X}$ is the design matrix, $\boldsymbol{\beta}_{\text{participant}}$ is the vector of regression coefficients of each participant, $\boldsymbol{\beta}$ is the vector of regression coefficients at the group-level, $\boldsymbol{\Sigma}$ is the covariance matrix of the regression coefficients, $\boldsymbol{\sigma}_{\text{method}}$ is a vector of standard deviations of the residuals defined for each artifact reduction method, and $\boldsymbol{\Omega}$ is the correlation matrix of the regression coefficients.

1.1.2 Within-Participants Effect Size Analysis

For the estimation of the posterior distribution of within-participants effect sizes of the difference between the contralateral and ipsilateral VEP responses, we implemented a model shown in S2. We modeled the standard deviation of the residuals with a two-level structure, where we estimated one standard deviation parameter for each participant and each method, pooled from five group-level standard deviations corresponding to the variability of residuals of each method within participants.

$$
\begin{aligned}
y_{\text{i}} &\sim \text{Normal}(X_{\text{i}}\boldsymbol{\beta}_{\text{participant[i]}}, \boldsymbol{\sigma}_{\text{participant,method[i]}}) \\
\boldsymbol{\beta}_{\text{participant}} &\sim \text{Normal}(\boldsymbol{\beta}, \boldsymbol{\Sigma}) \\
\boldsymbol{\beta} &\sim \text{Normal}(0, 10) \\
\boldsymbol{\Sigma} &= \text{var}(\boldsymbol{\beta})\ \boldsymbol{\Omega}\ \text{var}(\boldsymbol{\beta}) \\
\text{var}(\boldsymbol{\beta}) &\sim \text{Half-Cauchy}(0, 2.5) \\
\boldsymbol{\Omega} &\sim \text{LKJcorr(2)} \\
\boldsymbol{\sigma}_{\text{participant,method}} &\sim \text{Half-Normal}(\sigma_{\text{method}}, \sigma_{\boldsymbol{\sigma}}) \\
\boldsymbol{\sigma}_{\text{method}} &\sim \text{Half-Cauchy}(0, 2.5) \\
\sigma_{\boldsymbol{\sigma}} &\sim \text{Half-Cauchy}(0, 2.5),
\end{aligned}
\tag{S2}
$$

where y is the outcome variable, $\boldsymbol{X}$ is the design matrix, $\boldsymbol{\beta}_{\text{participant}}$ is the vector of regression coefficients of each participant, $\boldsymbol{\beta}$ is the vector of regression coefficients at the group-level, $\boldsymbol{\Sigma}$ is the covariance matrix of the regression coefficients, $\boldsymbol{\sigma}_{\text{method, participant}}$ is a vector of standard deviations of the residuals of each artifact reduction method and participant, $\boldsymbol{\sigma}_{\text{method}}$ is the group-level estimate of residuals of each method, $\sigma_{\boldsymbol{\sigma}}$ is the standard deviation of the group-level method standard deviations, and $\boldsymbol{\Omega}$ is the correlation matrix of the regression coefficients.

1.1.3 Across-Participants Effect Size Analysis and SNR on Participant Averages

Across-participants effect sizes of the contralateral–ipsilateral difference of the VEP responses and SNR on VEP participant-averages were estimated for each method separately. The inference was conducted with a single-level normal linear regression model S3.

$$
\begin{aligned}
y_{\text{i}} &\sim \text{Normal}(\boldsymbol{\mu}_{\text{method}}, \boldsymbol{\sigma}_{\text{method}}) \\
\boldsymbol{\mu}_{\text{method}} &\sim \text{Normal}(0, 10) \\
\boldsymbol{\sigma}_{\text{method}} &\sim \text{Half-Cauchy}(0, 2.5),
\end{aligned}
\tag{S3}
$$

where y is the outcome variable, $\boldsymbol{\mu}_{\text{method}}$ is the vector of mean signal-to-noise ratio/mean VEP difference of each method, and $\boldsymbol{\sigma}_{\text{method}}$ is the vector of standard deviations of signal-to-noise ratio/mean VEP difference of each method.

1.1.4 VEP Correlations

The correlation analysis of grand-average VEP responses for each of the four stimuli was estimated from the covariance of the bivariate Student's t-distribution, which is more robust to outliers than the normal distribution as assumed when computing Pearson correlation coefficients (Baez-Ortega, 2018; Bååth, 2013). Correlations were estimated with the model S4.

$$\begin{aligned}
\boldsymbol{y}_{\mathrm{i}} &\sim \mathrm{t}_{\nu}(\boldsymbol{\mu}, \boldsymbol{\Sigma}) \\
\boldsymbol{\Sigma} &= \begin{bmatrix} \sigma_1^2 & \sigma_1\sigma_2\rho \\ \sigma_1\sigma_2\rho & \sigma_2^2 \end{bmatrix} \\
\sigma_1, \sigma_2 &\sim \text{Half-Cauchy}(0, 2.5) \\
\boldsymbol{\mu} &\sim \text{Normal}(0, 100) \\
\nu &\sim \Gamma(2, 0.1),
\end{aligned} \tag{S4}$$

where y_{i} is a set of two two-dimensional vectors, $\boldsymbol{\Sigma}$ is the covariance matrix, σ_1 and σ_2 are the standard deviations of each input vector, $\boldsymbol{\mu}$ is the vector of means, ρ is the correlation coefficient, and ν is the degrees of freedom parameter.

1.2 Carbon-Wire Loop System

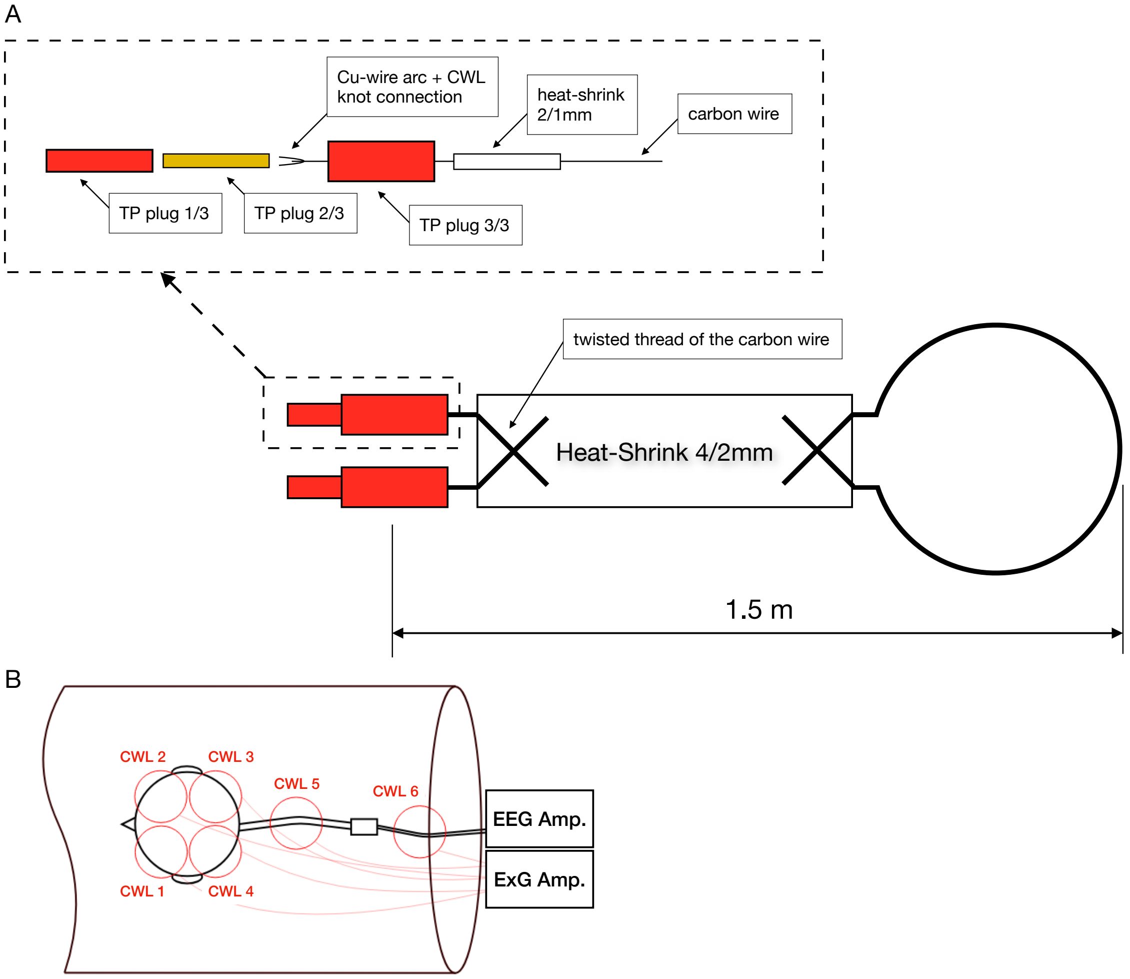

C

Required Materials

	1 CWL	6 CWL
**Insulated carbon wires [m]**	3	18
**Touch-proof plugs**	2	12
**Heat-shrink tubing 4/2mm [m]**	1.5	9
**Heat-shrink tubing 2/1mm [m]**	0.2	1.2
**Solid core Cu wire [m]**	0.05	0.3

Figure S1. Carbon-Wire Loop Construction and Materials. (A) A single carbon-wire loop (CWL) construction. **(B)** CWL system consisted of six CWLs connected to a bipolar amplifier (BrainAmp ExG, Brain Products GmbH, Gilching, Germany) with touch-proof (TP) plugs. Four CWLs were attached to the EEG cap and two CWLs to the leads connecting the cap to the amplifier. **(C)** A list of required materials for the construction of a single CWL and the entire set of six CWLs. The CWL system was designed according to van der Meer et al. (2016).

2 SUPPLEMENTARY ANALYSES

2.1 Motion-Induced Spectral Activity Analysis

Motion-induced spectral activity in the three frequency bands (alpha: 8–12 Hz, beta: 15–25 Hz, and gamma: 70–80 Hz) was estimated with the model shown in S5.

$$\begin{aligned} y_{\text{i}} &\sim \text{Normal}(\boldsymbol{\mu}_{\text{participant[i]}}, \sigma) \\ \boldsymbol{\mu}_{\text{participant}} &\sim \text{Normal}(\mu_{\mu}, \sigma_{\mu}) \\ \mu_{\mu} &\sim \text{Normal}(0, 5) \\ \sigma_{\mu} &\sim \text{Half-Cauchy}(0, 2.5) \\ \sigma &\sim \text{Half-Cauchy}(0, 2.5), \end{aligned} \tag{S5}$$

where y is the outcome variable, the mean power spectral density difference between finger-tapping and rest periods (ΔPSD), $\boldsymbol{\mu}_{\text{participant}}$ is the vector of ΔPSDs of each participant, μ_{μ} and σ_{μ} are the group-level mean and standard deviation parameters (hyperparameters of $\boldsymbol{\mu}_{\text{participant}}$), respectivelly, and σ is the standard deviation of the residuals on the first level.

The group-level posterior mean of the ΔPSD of the average signal of all four CWLs attached to participants' heads was estimated as (i) 0.501 dB/Hz (95% interval: 0.152–0.837 dB/Hz) for the alpha band (8–12 Hz), (ii) 0.595 dB/Hz (95% interval: 0.157–1.07 dB/Hz) for the beta band (15–25 Hz), and (iii) -0.0513 dB/Hz (95% interval: -0.311–0.211 dB/Hz) for the gamma band (70–80 Hz).

3 SUPPLEMENTARY FIGURES

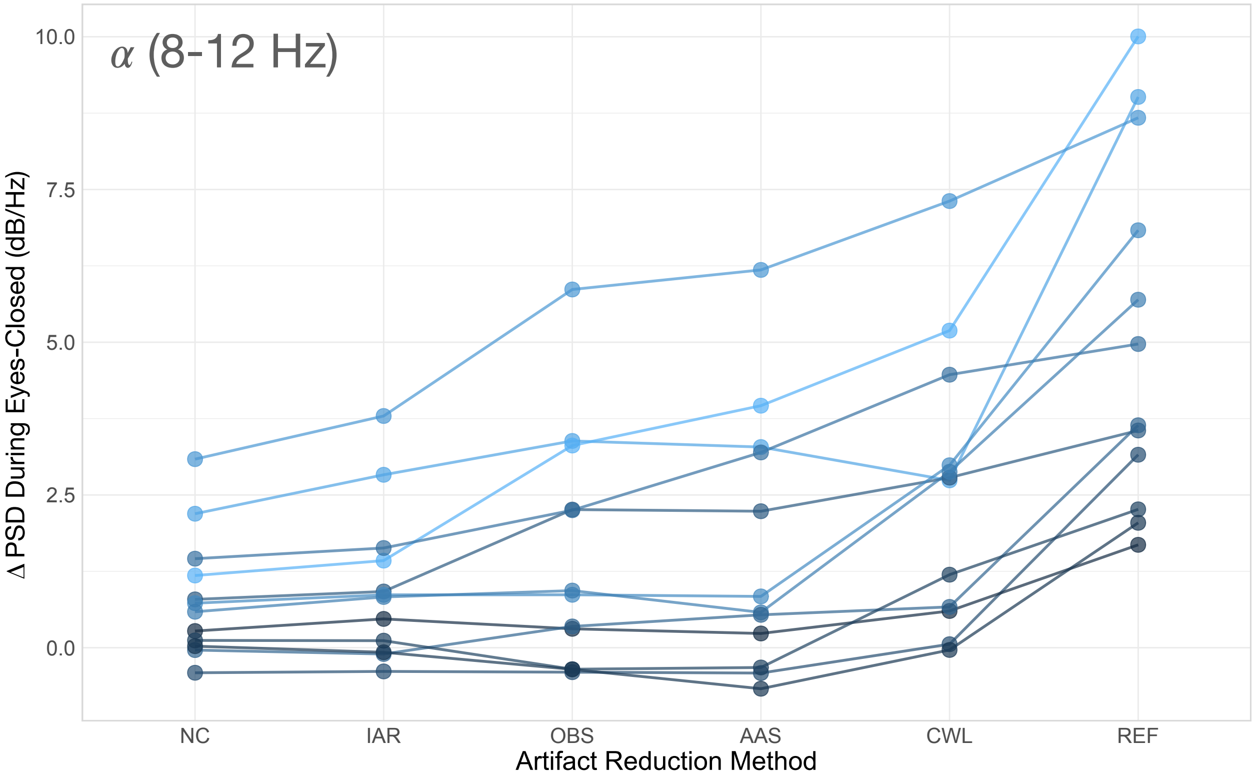

Figure S2. Resting-State Power Spectral Density Changes. Changes in mean power spectral density (ΔPSD) between the eyes-open (EO) and eyes-closed (EC) blocks in the resting state task for every participant and every dataset. **NC** – non-corrected signal, **IAR** – data with the imaging artifact reduced, **OBS** – data with IAR and reduced BCG artifacts with optimal basis set, **AAS** – data with IAR and reduced BCG artifacts with average artifact subtraction, **CWL** – data with IAR and carbon-wire loop artifact correction, **REF** – data collected outside of the MRI environment

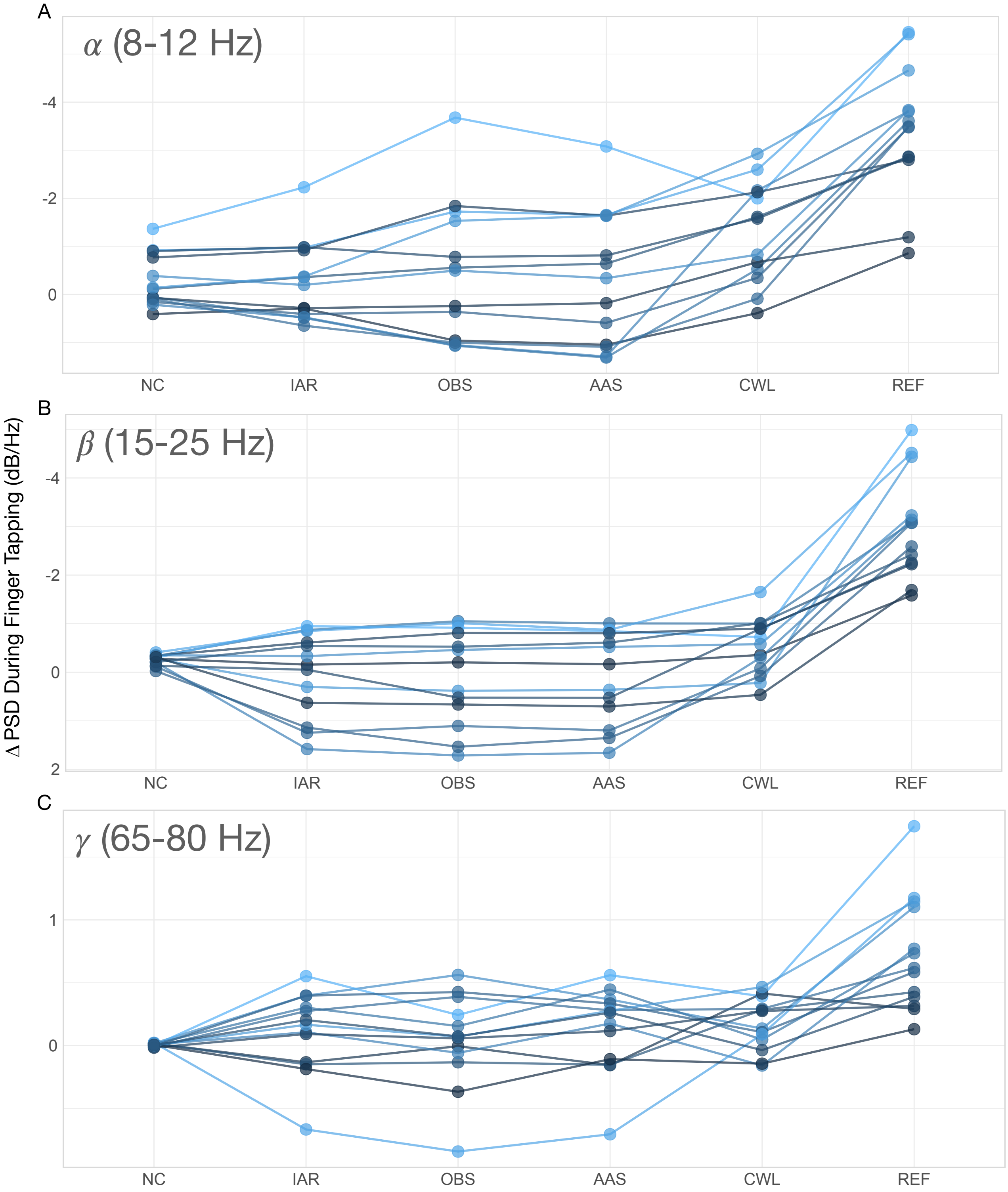

Figure S3. Finger Tapping Power Spectral Density Changes. Changes in mean power spectral density (ΔPSD) during finger tapping for every participant and every dataset in the **(A)** alpha, **(B)** beta and **(C)** gamma band. **NC** – non-corrected signal, **IAR** – data with the imaging artifact reduced, **OBS** – data with IAR and reduced BCG artifacts with optimal basis set, **AAS** – data with IAR and reduced BCG artifacts with average artifact subtraction, **CWL** – data with IAR and carbon-wire loop artifact correction, **REF** – data collected outside of the MRI environment

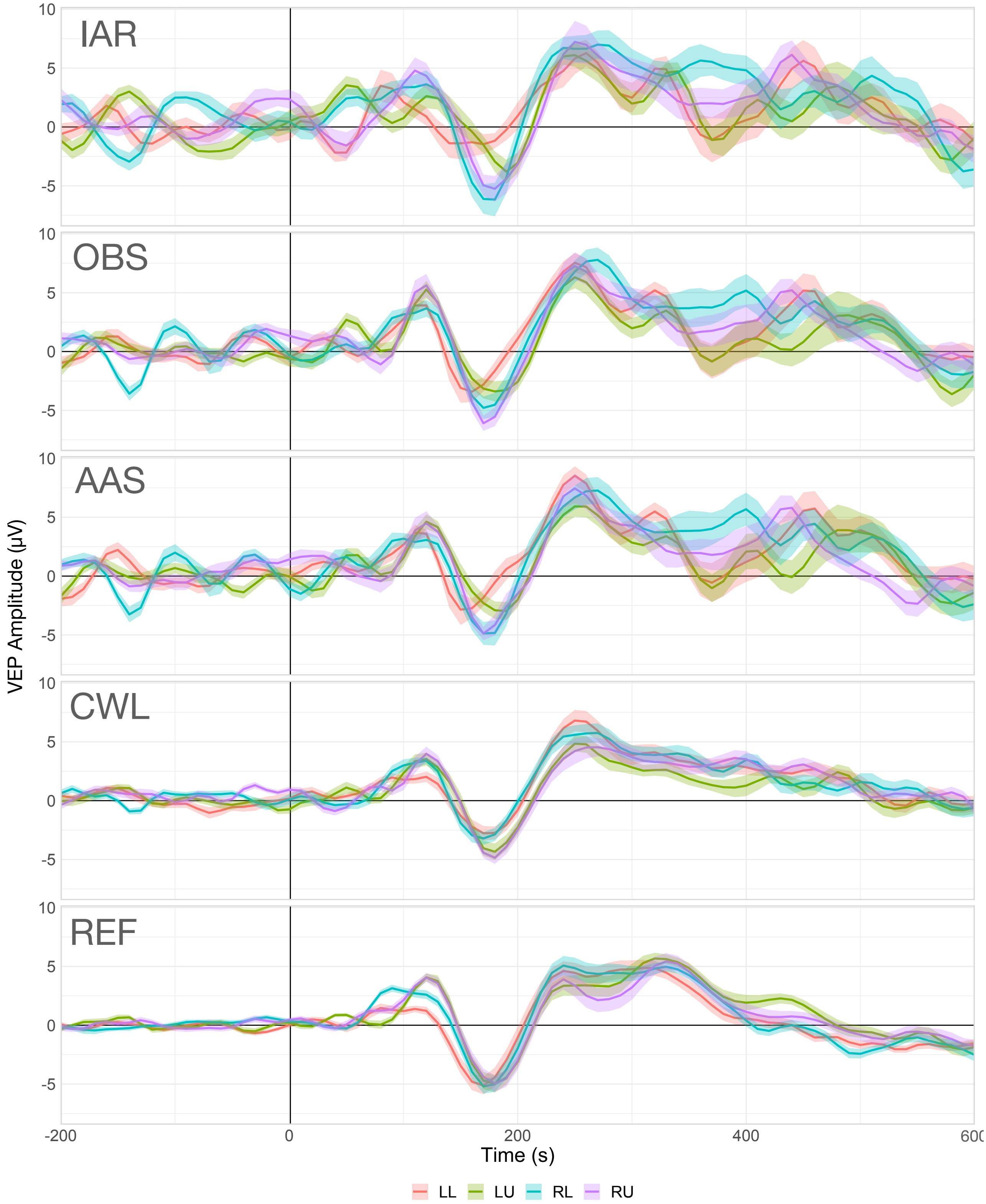

Figure S4. Grand-Average VEP. Grand-average visual-evoked potentials (VEP) from the checkerboard task for every data variant and every stimulus location (LL, LU, RL, RU). The ribbons indicate ± 2 between-participants standard errors. **LL** – left lower, **LU** – left upper, **RL** – right lower, **RU** – right upper, **IAR** – data with the imaging artifact reduced, **OBS** – data with IAR and reduced BCG artifacts with optimal basis set, **AAS** – data with IAR and reduced BCG artifacts with average artifact subtraction, **CWL** – data with IAR and carbon-wire loop artifact correction, **REF** – data collected outside of the MRI environment

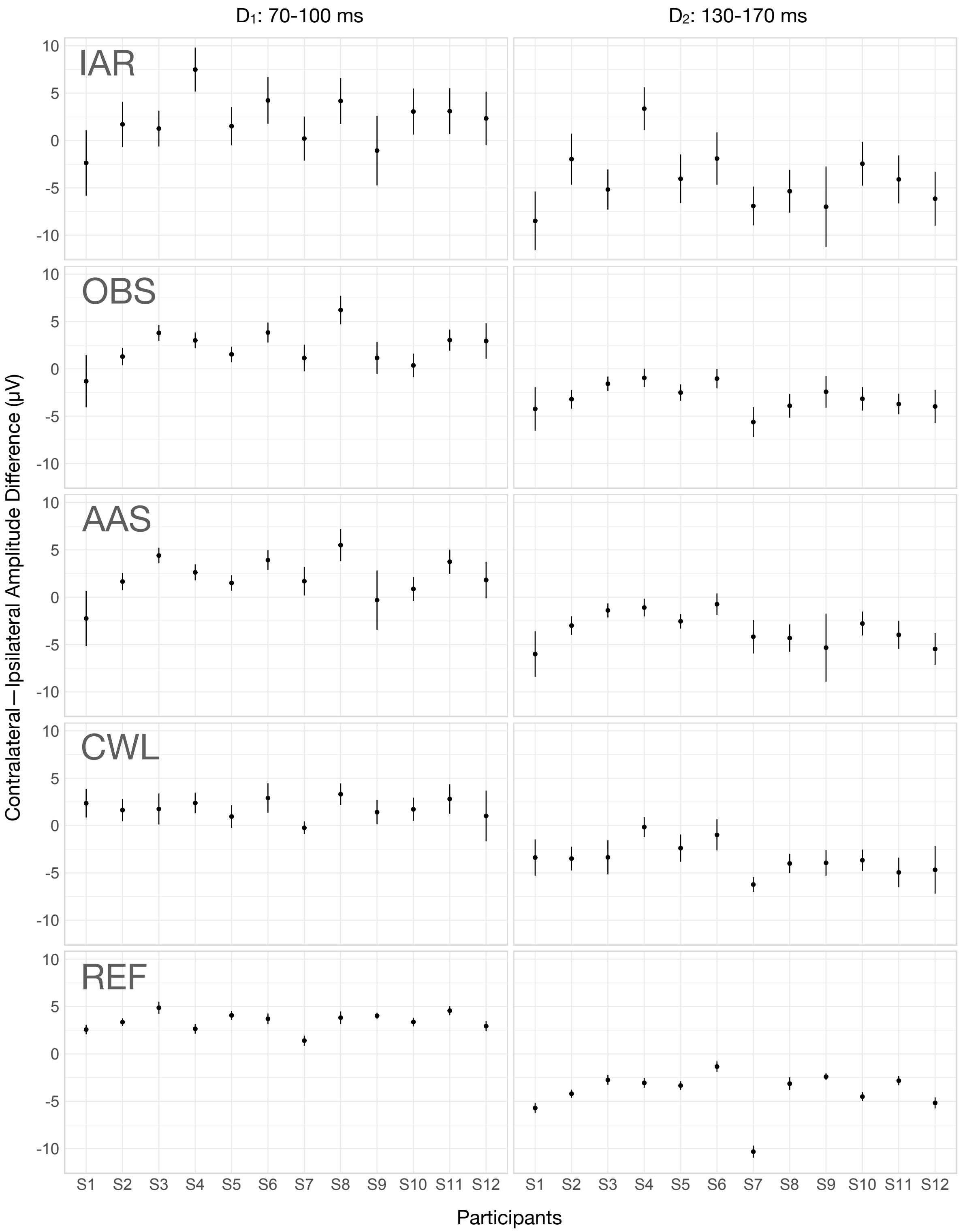

Figure S5. Within-Participants and Between-Participants Variability of the Mean Difference Wave Amplitude. Within-participants and between participants variability of the mean amplitude of the two examined peaks of the difference wave between the contralateral and ipsilateral participant-average VEP (D_1: 70–100 ms and D_2: 130–170 ms) for every participant and every data variant. **NC** – non-corrected signal, **IAR** – data with the imaging artifact reduced, **OBS** – data with IAR and reduced BCG artifacts with optimal basis set, **AAS** – data with IAR and reduced BCG artifacts with average artifact subtraction, **CWL** – data with IAR and carbon-wire loop artifact correction, **REF** – data collected outside of the MRI environment

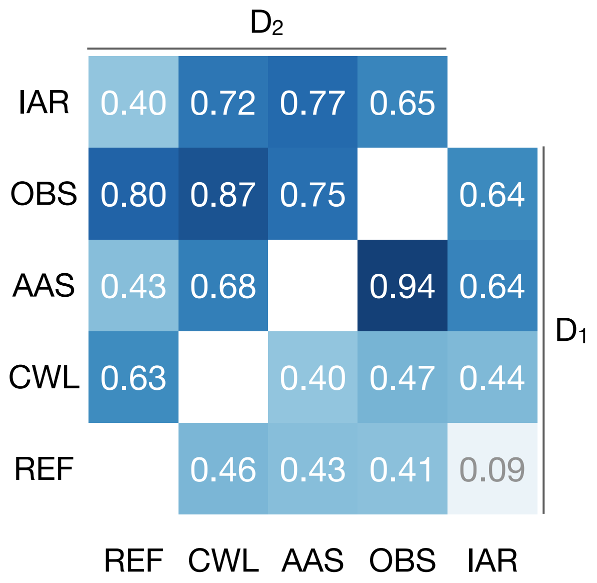

Figure S6. Correlations of Participants' Mean Difference Wave Peak Amplitudes. The correlation matrix of participant's mean difference wave peak amplitudes between data variants. The upper-left matrix contains correlations of the D_2 peak (130–170 ms), the lower-right matrix contains correlations of the D_1 peak (70–100 ms). **IAR** – data with the imaging artifact reduced, **OBS** – data with IAR and reduced BCG artifacts with optimal basis set, **AAS** – data with IAR and reduced BCG artifacts with average artifact subtraction, **CWL** – data with IAR and carbon-wire loop artifact correction, **REF** – data collected outside of the MRI environment

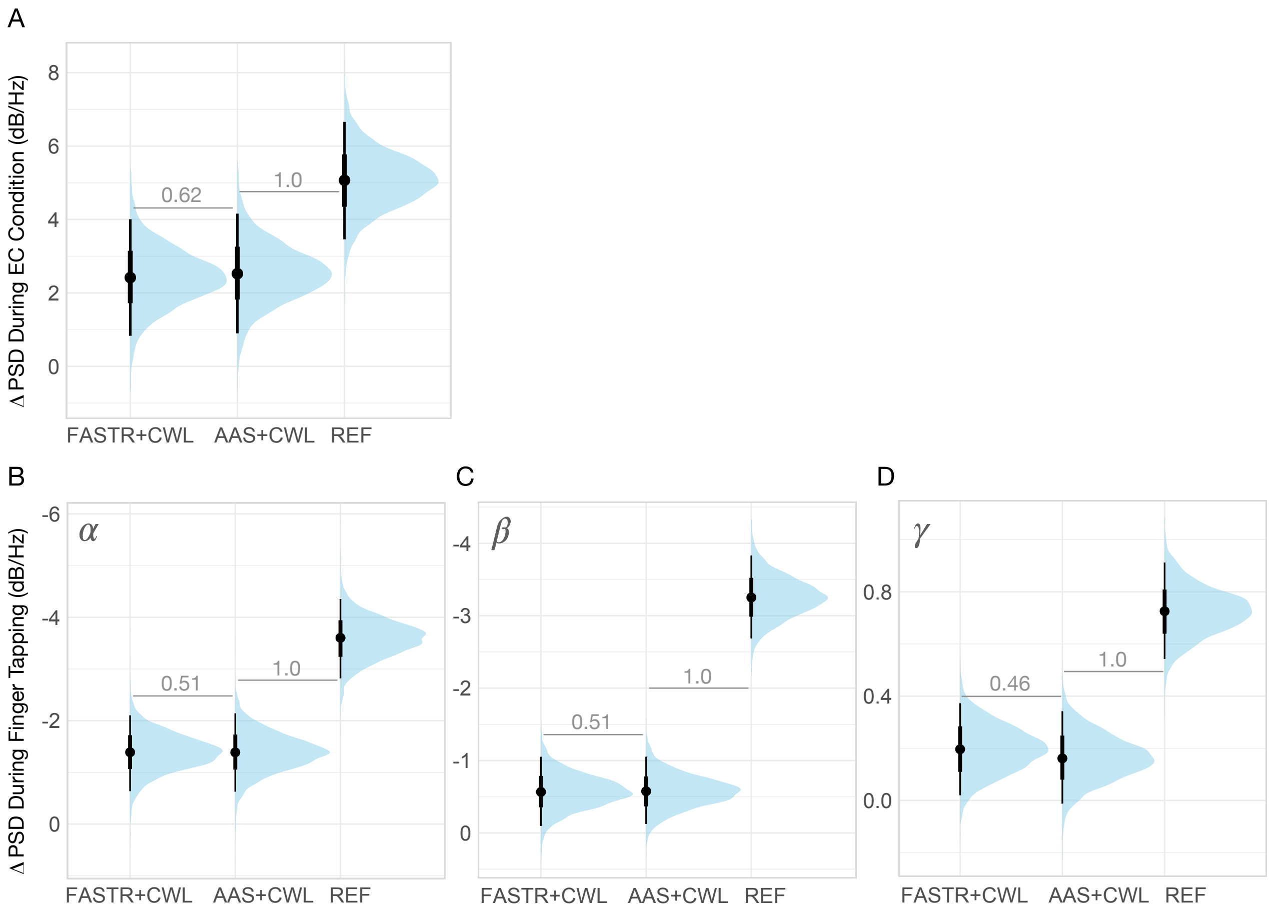

Figure S7. A Posteriori Spectral Analyses. (**A**) Posterior distributions of the group-level difference in mean PSD (ΔPSD) in the alpha band between EC and EO conditions for the FASTR+CWL, AAS+CWL, and REF datasets. The distributions are summarized with the median estimates and 66%-95% intervals. (**B**), (**C**) and (**D**) Posterior distributions of the group-level ΔPSD in the alpha, beta and gamma bands, respectively, for the FASTR+CWL, AAS+CWL, and REF datasets. The distributions are summarized with the median estimates and 66% and 95% intervals. Numbers between adjacent intervals depict the probability of the data variant on the right producing a larger ΔPSD than the one on the left, controlling for participant variability. **AAS+CWL** – data with imaging artifacts reduced using average artifact subtraction (AAS) and BCG artifacts reduced with carbon-wire loop (CWL) artifact correction, **FASTR+CWL** – data with imaging artifacts reduced using fMRI Artifact Slice Template Removal (FASTR) and BCG artifacts reduced with CWL artifact correction **REF** – data collected outside of the MRI environment

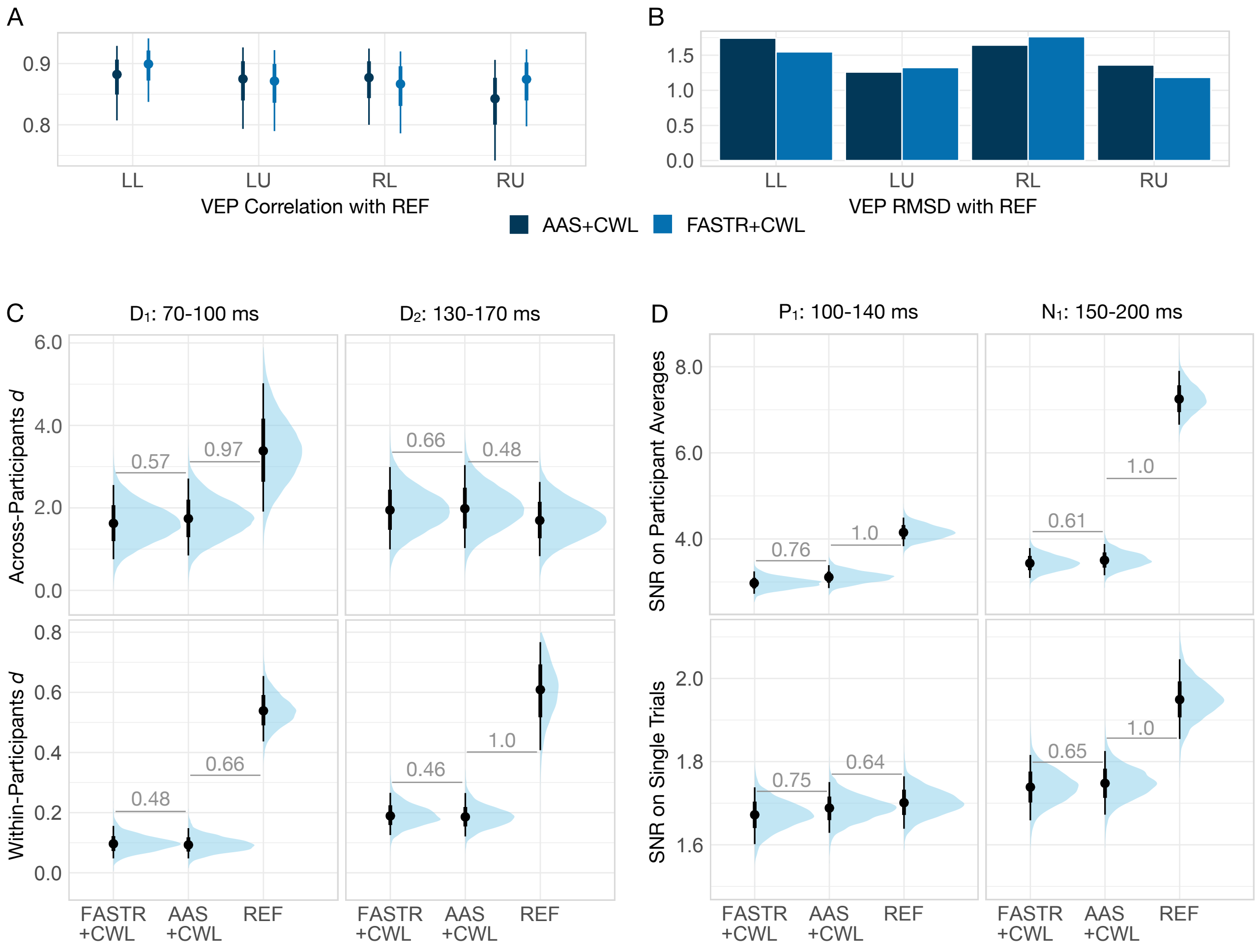

Figure S8. A Posteriori ERP Analyses. Posterior distributions of correlation coefficients **(A)** and root-mean-square deviations (RMSD) **(B)** between the grand-average VEP of FASTR+CWL and AAS+CWL data variants with the grand-average VEP of data collected outside of the MRI environment (REF) for each type of visual stimulus. Correlation distributions are represented with median estimates and 66%-95% intervals. **(C)** Posterior distributions of the across-participants effect size of both difference (D_1 and D_2) peaks (top row) and the posterior distribution of the group-average of within-participants effect sizes (bottom row). **(B)** Posterior distributions of SNR of both VEP peaks (P_1 and P_2) computed on participant averages (top row) and on single trials (bottom row). The distributions are summarized with median estimates and 66%-95% intervals. Numbers between adjacent intervals depict the probability of the method on the right producing a larger effect size/SNR than the method on the left, accounting for participant differences. **AAS+CWL** – data with imaging artifacts reduced using average artifact subtraction (AAS) and BCG artifacts reduced with carbon-wire loop (CWL) artifact correction, **FASTR+CWL** – data with imaging artifacts reduced using fMRI Artifact Slice Template Removal (FASTR) and BCG artifacts reduced with CWL artifact correction **REF** – data collected outside of the MRI environment

REFERENCES

Bååth, R. (2013). The Bayesian Counterpart of Pearson's Correlation Test. *Publishable Stuff - Rasmus Bååth's Research Blog*

Baez-Ortega, A. (2018). Bayesian robust correlation with Stan in R (and why you should use Bayesian methods). *in silico - Naïve thoughts on data*

Team, S. D. (2022). Stan Modeling Language Users Guide and Reference Manual

van der Meer, J. N., Pampel, A., Van Someren, E. J., Ramautar, J. R., van der Werf, Y. D., Gomez-Herrero, G., et al. (2016). Carbon-wire loop based artifact correction outperforms post-processing EEG/fMRI corrections—A validation of a real-time simultaneous EEG/fMRI correction method. *NeuroImage* 125, 880–894. doi:10/f74g39
